# Large-scale drug screening in iPSC-derived motor neurons from sporadic ALS patients identifies a potential combinatorial therapy

**DOI:** 10.1038/s41593-025-02118-7

**Published:** 2025-11-24

**Authors:** Christopher R. Bye, Elizabeth Qian, Katherine Lim, Maciej Daniszewski, Fleur C. Garton, Bảo C. Trần-Lê, Helena H. Liang, Tian Lin, John G. Lock, Duncan E. Crombie, Steven Morgan, Yi Hu, Samantha K. Barton, Lucy M. Palmer, Elvan Djouma, Saritha Kodikara, Kim-Anh Lê Cao, Thanuja Dharmadasa, Anjali K. Henders, Laura A. Ziser, Matthew C. Kiernan, Kevin Talbot, Merrilee Needham, Susan Fletcher, Paul Talman, Susan Mathers, Naomi R. Wray, Alex W. Hewitt, Alice Pebay, Bradley J. Turner

**Affiliations:** 1 The Florey Institute of Neuroscience and Mental Health, The University of Melbourne, Melbourne, Victoria Australia; 2 Centre for Eye Research Australia, Royal Victorian Eye and Ear Hospital, Melbourne, Victoria Australia; 3 Department of Anatomy and Physiology, The University of Melbourne, Melbourne, Victoria Australia; 4 Institute for Molecular Bioscience, The University of Queensland, Brisbane, Queensland Australia; 5 School of Biomedical Sciences, University of New South Wales, Sydney, New South Wales Australia; 6 Melbourne Bioinformatics, The University of Melbourne, Melbourne, Victoria Australia; 7 Department of Microbiology, Anatomy, Physiology and Pharmacology, La Trobe University, Melbourne, Victoria Australia; 8 Melbourne Integrative Genomics, School of Mathematics and Statistics, The University of Melbourne, Melbourne, Victoria Australia; 9 Neuroscience Research Australia, University of New South Wales, Sydney, New South Wales Australia; 10 Neurology Department, South Eastern Sydney Local Health District, Sydney, New South Wales Australia; 11 Nuffield Department of Clinical Neurosciences, University of Oxford, Oxford, UK; 12 Department of Neurology, Fiona Stanley Hospital, Perth, Western Australia Australia; 13 Centre for Molecular Medicine and Innovative Therapeutics, Murdoch University, Perth, Western Australia Australia; 14 Notre Dame University, Fremantle, Western Australia Australia; 15 Neurosciences Department, University Hospital Geelong, Geelong, Victoria Australia; 16 Calvary Healthcare Bethlehem Hospital Neuro-Consultancy, South Caulfield, Victoria Australia; 17 Queensland Brain Institute, The University of Queensland, Brisbane, Queensland Australia; 18 Menzies Institute for Medical Research, University of Tasmania, Hobart, Tasmania Australia; 19 Department of Surgery, Royal Melbourne Hospital, The University of Melbourne, Melbourne, Victoria Australia; 20 Perron Institute for Neurological and Translational Science, Perth, Western Australia Australia

**Keywords:** Amyotrophic lateral sclerosis, Induced pluripotent stem cells

## Abstract

Heterogeneous and predominantly sporadic neurodegenerative diseases, such as amyotrophic lateral sclerosis (ALS), remain highly challenging to model. Patient-derived induced pluripotent stem cell (iPSC) technologies offer great promise for these diseases; however, large-scale studies demonstrating accelerated neurodegeneration in patients with sporadic disease are limited. Here we generated an iPSC library from 100 patients with sporadic ALS (SALS) and conducted population-wide phenotypic screening. Motor neurons derived from patients with SALS recapitulated key aspects of the disease, including reduced survival, accelerated neurite degeneration correlating with donor survival, transcriptional dysregulation and pharmacological rescue by riluzole. Screening of drugs previously tested in ALS clinical trials revealed that 97% failed to mitigate neurodegeneration, reflecting trial outcomes and validating the SALS model. Combinatorial testing of effective drugs identified baricitinib, memantine and riluzole as a promising therapeutic combination for SALS. These findings demonstrate that patient-derived iPSC models can recapitulate sporadic disease features, paving the way for a new generation of disease modeling and therapeutic discovery in ALS.

## Abstract (web summary)

In this study, the authors generated iPSC lines from more than 100 sporadic ALS cases, which recapitulated key disease phenotypes and enabled large-scale drug screening, identifying a promising combination therapy of baricitinib, memantine and riluzole.

## Main

Amyotrophic lateral sclerosis (ALS) is a rapid and fatal disease defined pathologically by the selective degeneration of motor neurons in the brain and spinal cord. ALS results in progressive weakness and paralysis, impairing the ability to move, speak, swallow and breathe. Clinically heterogeneous, the causes of the disease are complex, multifactorial and poorly understood. Approximately 10% of patients present with a family history and/or harbor pathogenic variants in approximately 40 known ALS-causative genes, a condition referred to as familial ALS (FALS). The remaining 90% of cases have an unknown etiology and are traditionally classified as sporadic ALS (SALS). While important advancements have been made in identifying ALS-causative and risk factor genes, little progress has been achieved in determining the fundamental cause or disease mechanisms involved in SALS.

Therapeutic options for patients with SALS are limited. Riluzole remains the most widely prescribed treatment for ALS and is the only approved medication to extend life^1–3^. Edaravone and AMX0035 have also been granted regulatory approval in some countries; however, poor clinical evidence for their efficacy from real-world experiences or late-stage trials has limited the approval of edaravone in major jurisdictions and resulted in the removal of AMX0035 from the market. With current treatment and multidisciplinary care, the average life expectancy of persons diagnosed with ALS is less than 2.3 years^4^.

A major hurdle in understanding the etiology and pathophysiology of ALS, as well as in delivering effective therapies, has been the lack of animal or cell models for the predominant SALS^5–7^. In the absence of models of SALS, the field relies on systems that express gene mutations linked to rare forms of FALS, often with nonphysiological levels of expression and regulation. Since the discovery of riluzole over 30 years ago, more than 160 drugs shown to slow disease progression in FALS models have been clinically tested in patients with ALS. Few of these drugs have translated into clinically effective treatments in clinical trials^8^, and the pathophysiological relevance of those models to SALS has been increasingly questioned^9,10^. Developing pathophysiologically relevant models of SALS for drug development and preclinical testing remains a core challenge in ALS research, paralleling the experiences in other major neurodegenerative diseases, including Alzheimer’s disease and Parkinson’s disease.

The development of induced pluripotent stem (iPS) cells from patients initially promised to resolve this issue. Neurons generated from patient-derived iPS cells are genetically identical to the neurons in the central nervous system of the donor, providing a highly relevant human and pathophysiological model with endogenous gene expression and regulation^11,12^. Importantly, the generation of large-scale patient-derived iPS cell libraries provides the means to model sporadic diseases from across the patient population for the first time. Large-scale disease mapping with patient-derived neurons has the potential to greatly enhance our understanding of the molecular mechanisms driving these diseases and to assess drug efficacy more accurately and rapidly.

However, the potential of iPS cells to model SALS has not been realized to date. Despite the generation of iPS cell libraries on an unprecedented scale, along with extensive phenotypic, epigenetic, transcriptomic and proteomic analyses, no accepted model of SALS for drug development and testing has been generated^13^. Critically, no appropriately powered study has demonstrated reduced survival of SALS motor neurons compared to controls, which is a key pathological hallmark of ALS^14–22^. Pivotal phenotyping and drug screening studies, including those by Fujimori, Imamura and Wainger, which have recapitulated the spontaneous degeneration of SALS motor neurons, have included three or fewer control donors and have not conclusively demonstrated that a significant survival deficit is present in the underlying models^15–18,23,24^. Additionally, a large-scale analysis by the Answer ALS consortium using an iPS cell library with 1,000 lines has not reported survival deficits, TAR DNA-binding protein 43 (TDP-43) pathology or pharmacological rescue in SALS motor neurons. Concerningly, comprehensive transcriptomic, epigenetic and proteomic analyses from Answer ALS have consistently failed to demonstrate a separation between patients with SALS and healthy controls or to provide compelling evidence that the in vitro pathophysiological processes replicate those occurring in life in patients^23,25^. The implementation of SALS models lacking critical face validity risks producing experimental outcomes that may not accurately model the true disease state, making the question of whether sporadic disease can be recapitulated in patient-derived neurons a critically important and salient issue for neurodegenerative disease research.

To address this issue, we established a curated iPS cell library from 100 patients with SALS to capture the clinical, genetic and biological heterogeneity in the patient population. Using a rigorously optimized screening protocol, we conducted a population-wide phenotypic, transcriptional and pathological assessment of SALS motor neurons. Compared to motor neurons from healthy controls, those derived from patients with SALS exhibited impaired survival and accelerated neurite degeneration, which correlated with the survival of the donor. Transcriptional profiling of SALS motor neurons identified significant differential expression and generated a disease profile consistent with postmortem spinal cord tissues from patients with ALS. Pharmacological testing with the SALS model reproduced the efficacy of riluzole, rescuing motor neuron survival and reversing both electrophysiological and transcriptomic abnormalities. Implementing the approach for drug screening, we reassessed the efficacy of more than 100 drugs that have undergone clinical trials for ALS. Less than 5% of the tested drugs rescued motor neuron survival across SALS donors, reflecting the clinical trial outcomes for these drugs in patients. Combinatorial testing of the three effective drugs (riluzole, memantine and baricitinib) demonstrated that combinations of these drugs significantly increased the survival of SALS motor neurons, representing the first therapeutic candidates identified and validated across SALS donors to encompass the heterogeneity in drug efficacy within the SALS patient population. These findings provide compelling evidence that SALS can be modeled in patient-derived motor neurons and demonstrate their value for disease modeling, preclinical testing and drug screening for ALS.

## Results

### ALS iPS cell library generation

**Fig. 1:**
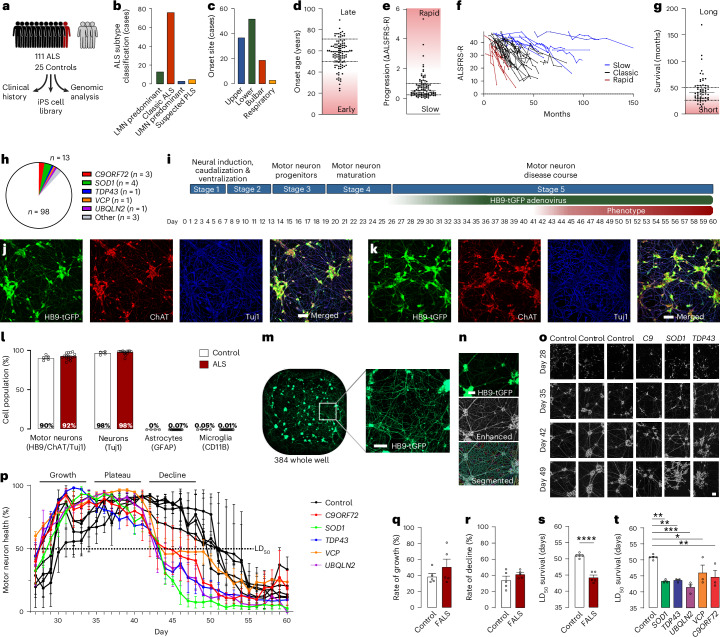
ALS iPS cell library generation and longitudinal live-cell phenotyping of motor neurons. **a**, Schematic of the iPS cell library donor composition and workflow. Each human figure represents approximately ten donors. **b**, Clinical subtype classification of ALS donors according to upper and lower motor neuron involvement. **c**–**g**, Key measures of clinical heterogeneity in the disease course of ALS donors: site of onset (*n* = 111) (**c**), age of onset (early onset <50 years and late onset >70 years, indicated on the scatter plot, *n* = 108) (**d**), median ALSFRS-R rate of decline (rapid progression >−1.5 per month and slow progression <−0.25 per month, indicated on the scatter plot) (**e**), longitudinal ALSFRS-R (**f**) and survival time (short survival <24 months and long survival >50 months, indicated on the scatter plot, *n* = 57) (**g**). **h**, Pathogenic or likely pathogenic variants in causal ALS genes previously reported in ALS cases identified in library donors using whole-genome sequencing (*C9ORF72*, *n* = 3 cases; *SOD1*, *n* = 4 cases; *TARDBP*/*TDP43*, *VCP* and *UBQLN2*, *n* = 1 case; other, *n* = 3 cases). **i**, Schematic of the optimized spinal motor neuron differentiation and phenotyping protocol. **j**,**k**, Representative immunohistochemistry images of terminally differentiated spinal motor neurons from control (**j**) and SALS (**k**) donors immunoreactive for HB9-tGFP, ChAT and Tuj1 at day 40. **l**, Quantification of spinal motor neurons (HB9^+^, ChAT^+^, Tuj1^+^), neurons (Tuj1^+^), astrocytes (GFAP^+^) and microglia (CD11B^+^) as a percentage of the total cells in culture at day 40 (mean ± s.e.m., *n* = 5 controls and 16 ALS donors). **m**, Representative whole-well live-cell image and magnified inset of viral HB9-tGFP reporter expression in spinal motor neurons. **n**, Quantification pipeline of HB9-tGFP expression from live-cell images showing the original image, enhanced image, and segmentation of cells and neurites conducted for all data points. **o**,**p**, Representative enhanced live-cell imaging of HB9-tGFP^+^ motor neurons (**o**) and longitudinal live-cell quantification of motor neuron neurite length (**p**) of healthy controls and FALS donors (expressed as a percentage of the maximum neurite length reached for each donor, averaged over replicate experiments; mean ± s.e.m., *n* = 3 independent experiments). The dotted line indicates a 50% decline in neurite length compared to its peak value, designated as LD_50_. **q**–**s**, Quantification of the neurite growth rate (*P* = 0.285) (**q**), rate of decline (*P* = 0.212) (**r**) and LD_50_ survival (**s**) of motor neurons from healthy controls and FALS donors (*P* = 0.0008, mean ± s.e.m., two-sided unpaired *t*-test, *n* = 5 donors per group). **t**, Quantification of the LD_50_ survival of motor neurons from healthy controls and FALS donors (mean ± s.e.m., one-way analysis of variance (ANOVA) with Holm–Šídák’s multiple comparisons test, main effect *P* = 0.0008, *SOD1* *P* = 0.002, *TDP43* *P* = 0.002, *UBQLN2* *P* = 0.0003, *VCP* *P* = 0.011, *C9ORF72* *P* = 0.005, *n* = 3). *P < 0.05, **P < 0.01, ***P < 0.001, ****P < 0.0001. Scale bar (**j**, **k** and **m**–**o**), 100 µm. LMN, lower motor neuron; UMN, upper motor neuron.

**Extended Data Fig. 1:**
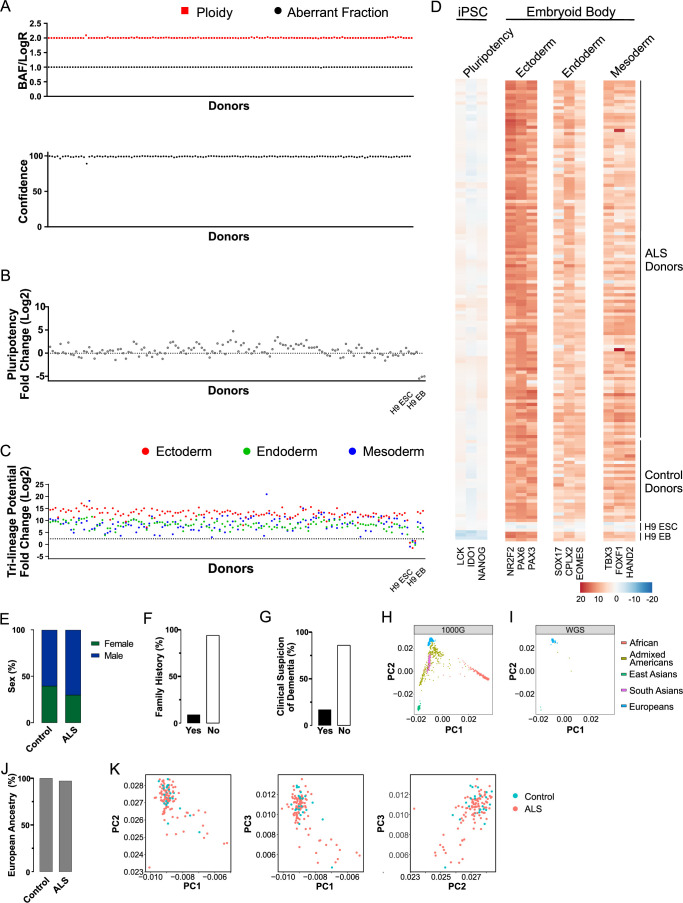
iPSC library line validation, clinical information and ancestry analysis. **a** Virtual karyotyping BAF/LogR plot and confidence score of genomic integrity for iPSC lines ordered by donor ID. **b** Quantification of pluripotency gene expression in iPSCs and **c** lineage gene expression for ectoderm, endoderm and mesoderm in differentiated embryoid bodies as an average of 3 genes per group (pluripotency/lineage) and **d** individual gene expression represented in a heatmap ordered by donor ID. **e** Donor demographic information plotting the sex of control and ALS donors, **f** percentage of ALS donors with a first degree relative with ALS and **g** percentage of ALS donors with a clinical suspicion of dementia (n=136 donors). **h** Genetic analysis (WGS) principal components plots (PC1-PC2) of the 1000G population and **i** the predicted matched ancestry using mean and standard deviation the sample cohort (n=136 donors). **j** Predicted European ancestry of control and ALS donors (expressed as % of cohort). **k** Principal components plots (PC1-PC3) restricted to donors with European ancestry population in the cohort (n=127 donors), disease status highlighted. Abbreviations: ALS; Amyotrophic Lateral Sclerosis, BAF; B-Allele frequency, EB; embryoid body, ESC; embryonic stem cell.

To facilitate large-scale ALS disease mapping and drug discovery across the heterogeneous patient population, an iPS cell library was derived from 100 patients with ALS who had no family history of the disease, 11 suspected monogenic cases and 25 healthy donors with no history of neurodegenerative diseases as controls (Fig. 1a). Fibroblasts were isolated from skin biopsy specimens from the donors and reprogrammed with nonintegrating episomal vectors using an automated robotics platform to maximize output and uniformity^26^. All lines were subjected to rigorous quality control testing^27^, including confirmation of genomic integrity, pluripotency and trilineage potential (Extended Data Fig. 1a–d). Patient donors were clinically assessed by a specialist ALS clinician and designated an ALS subtype classification based on upper and lower motor neuron involvement. Among the donors, 13 cases were classified as lower motor neuron-predominant ALS (including flail limb syndrome), 76 as classic ALS, 3 as upper motor neuron-predominant ALS and 5 as suspected primary lateral sclerosis (PLS) (Fig. 1b). The key measures of disease course—site of onset (Fig. 1c), onset age (Fig. 1d), disease progression measured by the median rate of decline based on the revised ALS functional rating scale (ALSFRS-R) (Fig. 1e,f), and survival time (Fig. 1g)—confirmed a clinically heterogeneous donor population, consistent with the general ALS patient population (Extended Data Fig. 1e–g). Whole-genome DNA sequencing in control and ALS cases established a predominant European ancestry for 95% of the donors (Extended Data Fig. 1h–k) and identified pathogenic or likely pathogenic variants or expansions in causal ALS genes in ten ALS donors (Fig. 1h and Supplementary Tables 1 and 2). Disease-causing expansions and variants in two related diseases (spinal and bulbar muscular atrophy or spinal muscular atrophy with progressive myoclonic epilepsy) were also identified in three ALS donors, and the presence of *C9ORF72* and *AR* expansion repeats was confirmed in both donor blood and iPS cell lines (Supplementary Table 2). ALS donors with no family history or causal variants identified were classified as having SALS (*n* = 98).

### Longitudinal live-cell imaging pipeline to assess motor neuron health

**Extended Data Fig. 2:**
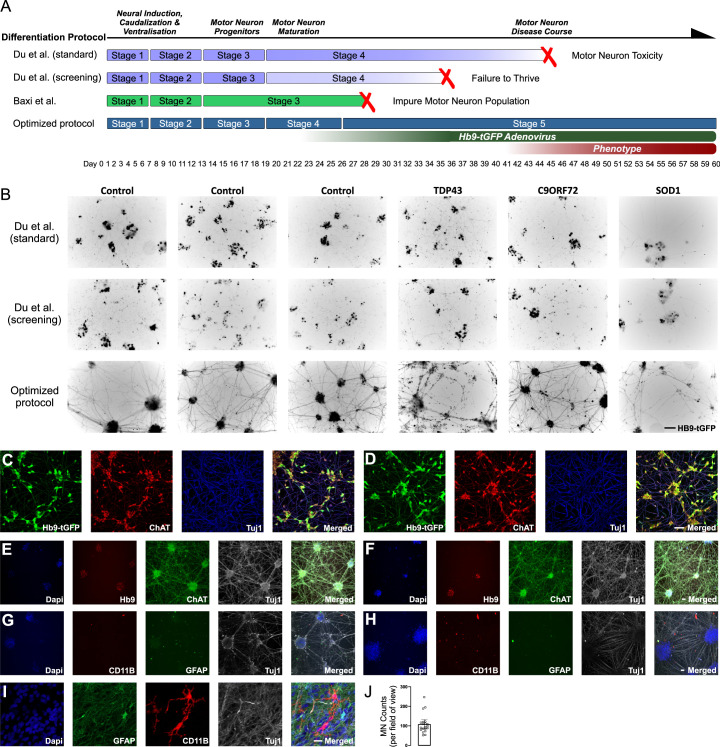
Motor neuron differentiation protocol optimization and cell characterization. **a** Schematic of tested spinal motor neuron differentiation and phenotyping protocols relative to the final optimised 5-stage protocol indicating stop/go decision points. **b** Images of motor neuron health at Day 40 of culture (Hb9-tGFP+ motor neuron clusters and neurites at 20x). Representative immunohistochemistry of terminally differentiated spinal motor neurons from **c** control and **d** SALS donors immunoreactive for HB9-tGFP, ChAT and Tuj1 at day 40. Representative immunohistochemistry from **e** control and **f** SALS donor showing motor neuron markers (HB9, ChAT and Tuj1) in terminally differentiated spinal motor neurons at day 40. Representative immunohistochemistry from **g** control and **h** SALS donor showing astrocyte (GFAP), microglia (CD11B) and Neuronal (Tuj1) markers in terminally differentiated spinal motor neurons at day 40. **i** Positive control for E and F showing GFAP, CD11B and Tuj1 immunolabelling in human organoids containing the 3 respective cell populations. **j** Quantification of motor neuron number (Hb9+, ChAT+, Tuj1+) per field of view (3 fields of view captured per well) used in turboGFP neurite length quantification (Day 43 of culture, mean ± 95% CI, n=36 donors). Scalebar B 100µm, C-D 100µm, E-H 200µm, I 20µm. Abbreviations: MN; motor neuron, tGFP; turboGFP.

Without a genuine pathological phenotype reflecting the clinical vulnerability of patients in life, iPS cell libraries hold little value for ALS research. We developed a robust motor neuron differentiation and phenotyping pipeline capable of modeling motor neuron degeneration, a key pathological hallmark of ALS and an important readout in the identification of effective therapeutics. A five-stage protocol was adapted from a well-established spinal motor neuron differentiation protocol^28^ with extensively optimized maturation and screening conditions capable of discriminating between healthy control and diseased motor neurons (Fig. 1i). Relative to other tested protocols (Extended Data Fig. 2a,b and Supplementary Table 3), our protocol generated consistently high-purity cultures of mature motor neurons displaying extensive neurite networks (Fig. 1j,k and Extended Data Fig. 2c,d). Implementing highly stringent quantification criteria^12^, 92.44 ± 1.66% (mean ± s.e.m.) of cells were defined as motor neurons, coexpressing choline acetyltransferase (ChAT), motor neuron and pancreas homeobox 1 (MNX1/HB9) and β-tubulin III (Tuj1) (Fig. 1l and Extended Data Fig. 2e,f). Additionally, the cultures contained 97.66 ± 0.99% Tuj1^+^ cells (neurons), 0.12 ± 0.01% GFAP^+^ cells (astrocytes) and 0.04 ± 0.02% CD11B^+^ cells (microglia) (Fig. 1l and Extended Data Fig. 2g–i). No differences in the proportions of these four cell types were detected between control and ALS donors. The highly enriched spinal motor neuron cultures provide a valuable reductionist system for assessing the cell-autonomous effects of ALS.

To assess motor neuron health, cultures were monitored daily using live-cell imaging in conjunction with a virally delivered nonintegrating motor neuron-specific reporter HB9-turboGFP (HB9-tGFP) (Fig. 1m). Extensive clustering of mature neurons prohibited accurate large-scale cell quantification, prompting the development of an alternative, fully automated method for quantifying the neurite length of motor neurons (Fig. 1n). By quantifying total neurite length from an average of 327 motor neurons per well in a 384-well plate format (Extended Data Fig. 2j), the pipeline provided a rapid, robust and reproducible assessment of neuronal health. Combined with an automated liquid handling platform to minimize variability, this approach enabled large-scale high-throughput phenotyping and drug screening.

**Extended Data Fig. 3:**
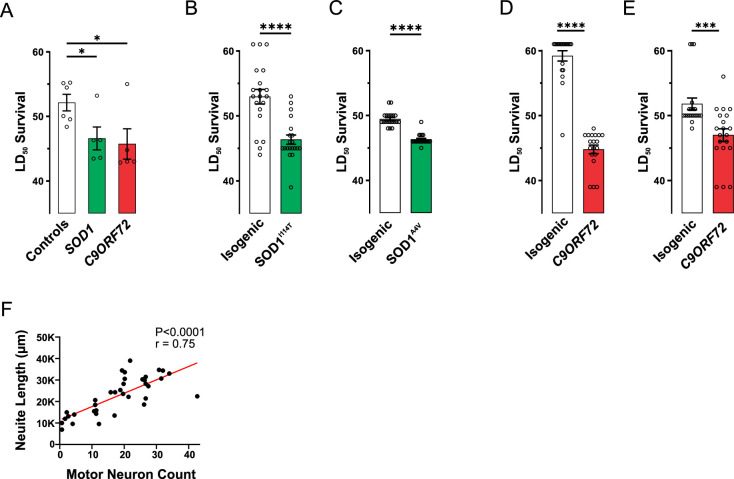
Longitudinal live-cell phenotyping of motor neurons from familial ALS donors. **a** Quantification of LD_50_ survival of motor neurons from healthy control and familial ALS donors harbouring variants in SOD1 (I114T, L145S, A96V, G94V, A4V) or expansions in C9ORF72 genes (mean ± SEM, one-way ANOVA with Holm-Sidak’s multiple comparisons test, main P=0.045, SOD1 P=0.047, C9ORF72 P=0.047, control n=6, FALS n=5 donors per group). **b-e** Quantification of LD_50_ survival of motor neurons from isogenic controls and familial ALS donors harbouring variants in SOD1 or expansions in C9ORF72 genes (mean ± SEM, two-sided unpaired t test, SOD1^I114T^ P<0.0001, SOD1^A4V^ P<0.0001, C9ORF72 P<0.0001, C9ORF72 P=0.0009, n= 20 wells/technical replicates per donor). **f** Spearman correlation analysis of turboGFP neurite length quantification and confocal turboGFP motor neuron quantification per well at Day 43 of culture (two-sided, n=36 donors). Abbreviations: LD_50_, Lethal day 50%.

To demonstrate the utility of the phenotyping pipeline, motor neuron health was assessed in five FALS donors and five healthy controls encompassing five ALS genes (*SOD1*, *TARDBP*/*TDP43*, *C9ORF72*, *VCP* and *UBQLN2*). Time course analysis of motor neuron neurite length across donors showed a consistent growth phase as the neurons matured and became more morphologically complex, followed by a plateau and a degeneration phase (Fig. 1o,p). Motor neurons from FALS donors did not show a significant difference in the rate of growth (percentage increase in neurite length over 48 h) compared to control donors, nor in the rate of decline (percentage decrease in neurite length over 48 h) (Fig. 1q,r). Motor neuron survival time was assessed by calculating the day on which the total neurite length of each donor dropped to 50% of its peak (lethal day 50% (LD_50_)), in an approach reminiscent of the lethal dose 50 measurement used in pharmacology. LD_50_ survival time (dotted line, Fig. 1p) was significantly reduced in FALS donors (*P* = 0.0008) by up to 8 days (Fig. 1s) compared to control donors, demonstrating that the phenotyping pipeline was capable of sensitive detection of ALS neurodegenerative phenotypes. Replication of the screen (*n* = 3 independent differentiations) identified a significant effect of ALS variants (*P* = 0.0008), with a decrease in LD_50_ survival in donors harboring *SOD1* (*P* = 0.002), *TDP43* (*P* = 0.002), *UBQLN2* (*P* = 0.0003), *VCP* (*P* = 0.011) and *C9ORF72* (*P* = 0.005) mutations (Fig. 1t). Additional replication across FALS donors harboring different variants in the same gene (Extended Data Fig. 3a) and between FALS and isogenic control lines (Extended Data Fig. 3b–e) also detected significant decreases in LD_50_ survival. To ensure that the quantification of LD_50_ survival in the automated assay accurately reflected motor neuron survival, live-cell imaging of neurite length and immunohistochemical quantification of HB9-tGFP^+^ motor neuron numbers from confocal images were conducted on the same cells on day 43. Motor neuron survival and neurite length in the cultures were significantly correlated (*P* < 0.0001, *r* = 0.75; Extended Data Fig. 3f), validating the use of automated neurite quantification as a direct readout of motor neuron health. Importantly, the culture conditions provoked an accelerated life cycle for motor neurons that induced spontaneous degeneration without the use of exogenous stressors, toxins or glial cell coculture (for example, astrocytes).

### Large-scale phenotypic screening of patient-derived SALS motor neurons

**Fig. 2:**
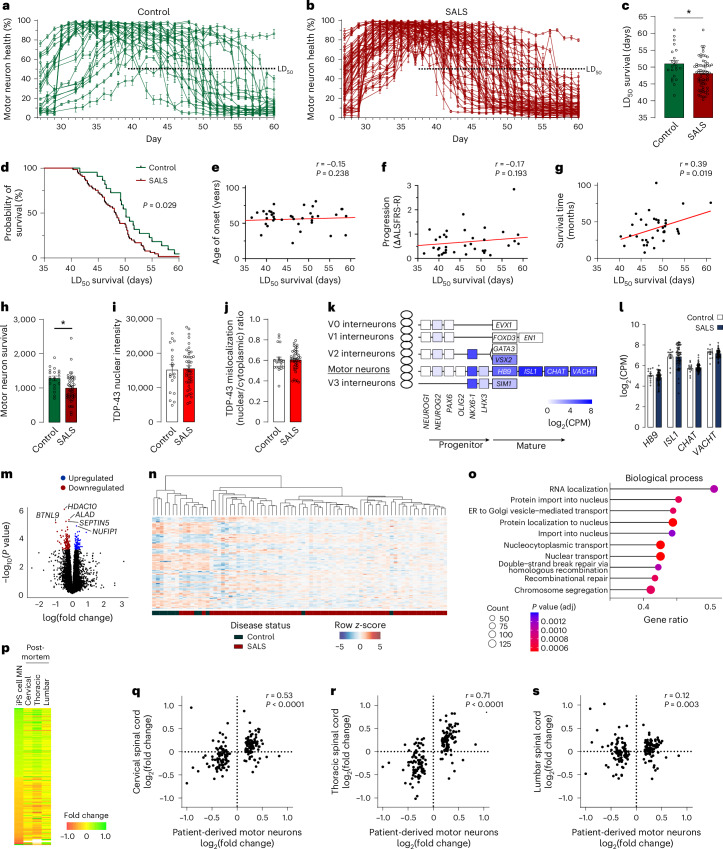
Longitudinal live-cell phenotyping of motor neurons. **a**,**b**, Longitudinal quantification of the total neurite length of HB9-tGFP^+^ motor neurons, from live-cell images of all healthy controls (**a**) and SALS donors (**b**) in the library producing quantifiable data (expressed as a percentage of the maximum neurite length for each donor, mean of six wells ± s.e.m., *n* = 22 controls and 65 SALS donors). **c**, Quantification of the LD_50_ survival of motor neurons from the healthy controls and SALS donors shown in **a** and **b** (mean ± s.e.m., two-sided unpaired *t*-test, *P* = 0.012, *n* = 22 controls and 65 SALS donors). **d**, LD_50_ probability of survival of healthy control donors compared to SALS donors (log-rank test, *n* = 22 controls and 65 SALS donors). **e**–**g**, Pearson’s correlation analysis of patient-derived motor neuron LD_50_ survival and available clinical data for donor age of onset (*n* = 65 donors) (**e**), donor rate of progression (change in ALSFRS-R, *n* = 62 donors) (**f**) and donor survival time (two-sided, *n* = 36 donors) (**g**). **h**–**j**, Quantification of ChAT-immunoreactive cell number (*P* = 0.013) (**h**), nuclear TDP-43 immunoreactivity (average intensity, *P* = 0.8316) (**i**) and ratio of nuclear TDP-43 intensity to cytoplasmic intensity (*P* = 0.990) (**j**) in a subset of healthy controls and SALS donors at day 42 (mean ± s.e.m., two-sided unpaired *t*-test, *P < 0.05, *n* = 19 controls and 44 SALS donors). **k**, Heatmap representing the expression (RNAseq counts) of developmental markers of dorsal ventral patterning in the ventral spinal cord at day 49 (*n* = 12 controls and 65 SALS donors). **l**, Expression (RNAseq counts) of markers of mature motor neurons in control and ALS donors (mean ± s.e.m., *HB9* *P* = 0.629, *ISL1* *P* = 0.999, *CHAT* *P* = 0.944, *VACHT* *P* = 0.944, two-sided unpaired *t*-test with Welch correction and Holm-Šídák’s multiple comparisons test, *n* = 12 controls and 65 SALS donors). **m**, Volcano plot representing fold changes and *P* values; 192 genes were identified as differentially expressed between controls and ALS donors (adjusted *P* < 0.05 highlighted, with the five most significant genes labeled, empirical Bayes moderated *t* statistics (limma–voom) with Benjamini–Hochberg multiple testing adjustment, *n* = 12 controls and 65 SALS donors). **n**, Hierarchical clustering and heatmap of differentially expressed genes (adjusted *P* < 0.05) identified between controls and SALS donors. **o**, Top 10 significantly overrepresented Gene Ontology gene set (GSEA) between controls and ALS donors (adjusted *P* < 0.01, Kolmogorov–Smirnov test with Benjamini–Hochberg multiple testing adjustment). **p**–**s**, Heatmap (**p**) and Spearman’s correlation analysis of patient-derived motor neuron disease profiles (192 differentially expressed genes) compared to cervical (**q**), thoracic (**r**) and lumbar (**s**) postmortem spinal cords (control versus SALS, log_2_(fold change)) sourced from Humphrey et al.^32^ (two-sided). *P < 0.05. adj, adjusted; CPM, counts per million; ER, endoplasmic reticulum; MN, motor neuron.

**Extended Data Fig. 4:**
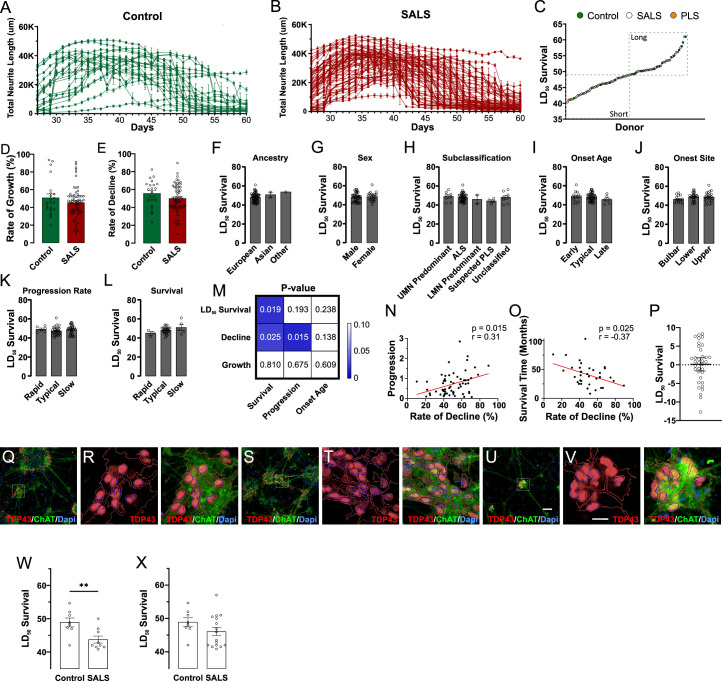
Phenotypic Screening of SALS motor neurons. **a-b** Longitudinal quantification of total Hb9-tGFP^+^ motor neuron neurite length from live cell images of all healthy control and sporadic ALS (SALS) donors in the library producing quantifiable data (total neurite length, mean ± SEM, control n=22 donors and SALS n=65 donors). **c** Representation of LD_50_ survival of motor neurons ranked from shortest to longest survival for all donors (control n=22 donors, SALS n=65 donors and suspected PLS n=4 donors). **d** Quantification of rate of growth and **e** rate of decline of motor neurons from healthy control and sporadic ALS donors (mean ± SEM, two-sided unpaired t test, n=22 control and 65 SALS donors). **f-l** Quantification of LD_50_ survival of motor neurons across demographic and clinical subtypes of SALS donors including all available clinical data (mean ± SEM, one-way ANOVA, ancestry, sex, ALS subtype classification and onset age n=65 donors, site of onset n=63 donors, disease progression n=62 donors, and clinical survival time n=36 donors). **m** Pearson’s correlation matrix showing P value of patient-derived motor neuron rate of growth, rate of decline and LD_50_ survival and the available clinical data for donor survival time (n=36 donors), rate of progression (change in ALSFRS-R, n=62 donors) and disease onset age (two-sided, n=65 donors). **n** Pearson’s correlation analysis of patient-derived motor neuron rate of decline and rate of progression (change in ALSFRS-R, two-sided, n=62 donors), and **o** patient-derived motor neuron rate of decline and clinical donor survival time (two-sided, n=36 donors). **p** Quantification of variation in LD_50_ survival across independent replicates, expressed as difference from original phenotypic screen data (mean ± 95% CI, one sample t-test, n=35 donors). Representative confocal immunohistochemistry images and magnified inserts of TDP43 immunolabeling in Chat positive motor neurons at D42 of culture in a **q-r** healthy control donor, **s-t** SALS donor and **u-v** familial ALS donor harbouring a variant in the TDP43 gene. Red traces indicate delineation of nucleus and cytoplasm used in quantification. **w** Quantification of LD_50_ survival of motor neurons from healthy control (LD_50_ 48-52, n=9 donors) and SALS donors (LD_50_ <46, n=8 donors) with known phenotypes (mean ± SEM, two-sided unpaired t test, P=0.008). **x** Quantification of LD_50_ survival of motor neurons from healthy control (LD_50_ 48-52, n=9 donors) and SALS donors with unknown phenotypes (n=15 donors) (mean ± SEM, two-sided unpaired t test). Scalebar Q,S,U 100µm, R,T,V 25µm. Abbreviation: ALSFRS-R; ALS functional rating scale - revised, LD50; Lethal day 50%, LMN; lower motor neuron, PLS; primary lateral sclerosis, SALS, sporadic ALS, tGFP; turboGFP, UMN; upper motor neuron.

Large-scale phenotypic screening using live-cell imaging was conducted on the entire iPS cell library with 74% of donors passing quality control criteria, revealing variability across control and SALS donors (Fig. 2a,b and Extended Data Fig. 4a–c). The LD_50_ survival time was significantly reduced in SALS donors compared to healthy controls (*P* = 0.012; Fig. 2c), providing the first evidence of accelerated neurodegeneration in SALS supported by sufficient numbers of control and SALS donors to provide appropriate statistical power^15–18,23^. Similar to FALS, the rate of motor neuron growth or decline did not differ significantly between healthy control and SALS donors (Extended Data Fig. 4d,e). Direct comparison of LD_50_ survival curves using the log-rank test demonstrated that survival in SALS donors was significantly reduced compared to healthy controls (*P* = 0.029), consistent with a reduced lifespan (Fig. 2d). Within SALS donors, no significant difference in LD_50_ survival was observed among donor demographic or clinical subtypes (Extended Data Fig. 4f–l).

To assess the ability of motor neurons derived from patients with SALS to model the clinical symptoms of ALS, the in vitro LD_50_ survival deficit was compared to the key clinical parameters: age of disease onset, progression rate (measured by the change in ALSFRS-R) and survival (Fig. 2e–g). A significant correlation between the LD_50_ survival of motor neurons and the survival time of patients was evident (*P* = 0.019, *r* = 0.39), supporting a link between patient-derived motor neurons and clinical prognosis. Additionally, a significant correlation was observed between the in vitro rate of decline and both clinical progression rate (*P* = 0.015, *r* = 0.31) and clinical survival time (*P* = 0.025, *r* = −0.37) (Extended Data Fig. 4m–o). The detection of significant correlations, despite the high variability in both the iPS cell technology and clinical data, was notable, with the moderate correlation coefficients reflecting this high variability. Thus, the LD_50_ survival of motor neurons derived from patients with SALS correlates with key aspects of the clinical vulnerability of patients in life.

To confirm that the LD_50_ survival deficit reflected a reduced survival of motor neurons in SALS donors, the number of ChAT-immunolabeled motor neurons was quantified in 45 SALS donors and 19 healthy control donors. Quantification was conducted on day 42, a time point at which the LD_50_ survival indicated prominent neuronal degeneration in SALS donors. Motor neuron numbers were significantly reduced in SALS donors compared to control donors (*P* = 0.013, 77.8%), confirming reduced motor neuron survival in patient-derived SALS motor neurons (Fig. 2h). In parallel, TDP-43 protein expression was assessed to identify evidence of TDP-43 dysregulation in the patient-derived motor neurons. TDP-43 immunolabeling remained predominantly nuclear in both control and SALS donors, with limited mislocalization or aggregation in the cytoplasm (Extended Data Fig. 4q–v). Quantification of TDP-43 intensity in motor neurons revealed no significant nuclear depletion or mislocalization of TDP-43 in SALS donors (Fig. 2i,j). Therefore, we conclude that patient-derived SALS motor neurons display reduced survival, a phenotype that occurs without obvious TDP-43 pathology in our system.

To support future studies seeking to phenotype SALS donors, small-scale phenotypic screens were conducted to determine the minimum number of donors required to detect an LD_50_ survival deficit. First, an experiment comparing SALS donors with known strong phenotypes (LD_50_ < 45, *n* = 9) and controls with typical phenotypes (LD_50_ ~ 50, *n* = 8) was conducted. Power analysis estimated that a sample size of nine donors per group was required (*α* = 0.05, *β* = 0.2), and a significant decrease in the LD_50_ survival of SALS donors was detected (Extended Data Fig. 4w; *P* = 0.008). A second experiment compared SALS donors with unknown phenotypes (*n* = 15) to controls with typical phenotypes (LD_50_ ~ 50, *n* = 8). Power analysis estimated that a larger sample size of 18 donors per group was required (*α* = 0.05, *β* = 0.2). Accordingly, no significant difference in the LD_50_ survival of SALS donors was detected (Extended Data Fig. 4x). These sample sizes will serve as important guides for future experimentation in SALS and other sporadic neurodegenerative diseases.

### Large-scale molecular profiling of patient-derived SALS motor neurons

**Extended Data Fig. 5:**
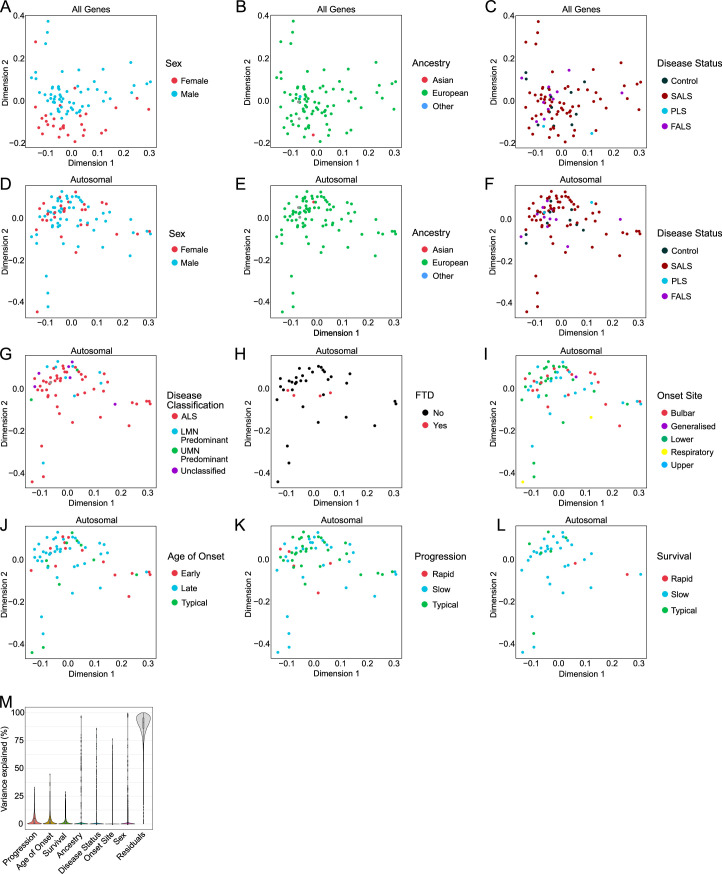
Principle components analysis of ALS motor neurons transcriptional profiles. **a-c** Principal components plots (PC1-PC2) of RNAseq profiling including all genes highlighting sex, ancestry and ALS disease status of donors (n=12 controls, 65 SALS, 2 PLS and 11 FALS donors). **d-l** Principal components plots (PC1-PC2) of RNAseq profiling restricted to autosomal genes only highlighting sex, ancestry, clinical subtypes, disease status, ALS subtype classification (n= 88 donors), FTD (n= 57 donors), site of onset (n= 86 donors), onset age (n= 86 donors), disease progression (n= 72 donors) and clinical survival time(n= 42 donors) including all available clinical data. **m** Variance partition analysis of RNAseq profiling of sex, ancestry, disease status (n= 88 donors), site of onset (n= 86 donors), onset age (n= 86 donors), disease progression (n= 72 donors) and clinical survival time (n= 42 donors) including all available clinical data. Abbreviation: FALS; familial ALS, LMN; lower motor neuron, PLS; primary lateral sclerosis, SALS, sporadic ALS, UMN; upper motor neuron.

To further assess the ability of patient-derived ALS motor neurons to model ALS pathogenesis, RNA sequencing (RNAseq) transcriptional profiling of in vitro motor neurons was compared to the transcriptional profiles of postmortem spinal cord tissues from patients with SALS. Profiling of in vitro motor neurons was conducted at the latest feasible time point at which donors retained viable cells (day 49). A total of 88 samples passed all quality control criteria, and the expression of developmental genes confirmed specific dorsoventral patterning in the cultures (Fig. 2k). Critically, consistent gene expression of the motor neuron markers *MNX1* (*HB9*), *ISL1*, *CHAT* and *SLC18A3* (*VACHT*) indicated the uniform generation of motor neurons across donors, which is essential for accurate transcriptional profiling (Fig. 2l). Principal component analysis revealed separation of donors based on sex, as previously reported^23,25^, but not by ethnicity or disease status (Extended Data Fig. 5a–c). Exclusion of sex chromosomes reversed the separation of donors by sex (Extended Data Fig. 5d). Plotting of autosomal genes only (Extended Data Fig. 5e–l) or variance partitioning analysis (Extended Data Fig. 5m) revealed no strong separation of donors by demographic or clinical parameters.

**Extended Data Fig. 6:**
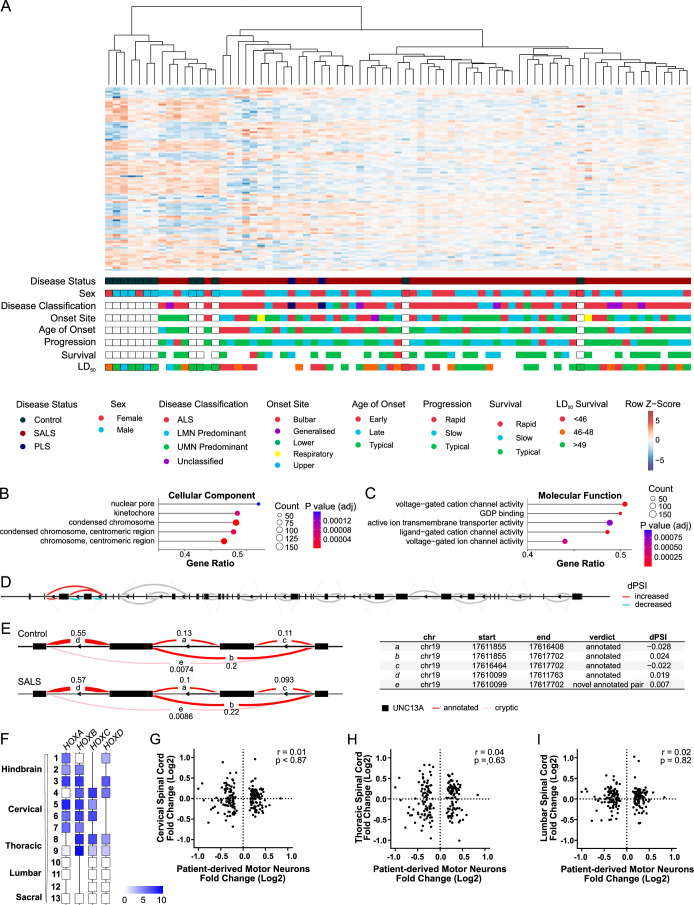
Clinical and *in vitro* transcriptional and splicing profiles of SALS motor neurons. **a** Hierarchical clustering and heatmap of differentially expressed genes (adjusted P value <0.05) identified between control and sporadic ALS donors with donor demographic (sex), clinical (disease status, ALS subtype classification, site of onset site, age of onset, disease progression, clinical survival time) and in vitro motor neuron phenotypic (LD_50_ survival) information (n=88 donors, available clinical data included). **b-c** Top 5 significantly overrepresented gene ontology gene sets (GSEA) between control and ALS donors (adjusted P value <0.01, Kolmogorov-Smirnov test with Benjamini–Hochberg multiple testing adjustment). **d** Gene level visualisation of differential splicing between SALS and control donors in UNC13A, with significant changes coloured. **e** Magnified visualisation and table of significant differential splicing between SALS and control donors in UNC13A showing annotated (dark) and cryptic (light) exon expression. **f** Heatmap representing the expression (RNAseq counts) of developmental markers of rostro caudal patterning in the ventral spinal cord at Day 49 (n=12 control and 65 SALS donors) **g-i** Spearman’s correlation analysis of randomised patient-derived motor neuron disease profile (192 differentially expressed genes, adjusted P value <0.05, n=12 control and 65 SALS donors) compared to cervical, thoracic and lumbar post-mortem spinal cord (control vs sporadic ALS, log2 fold change) sourced from Humphrey et al (two-sided). Abbreviation: ALS; Amyotrophic Lateral Sclerosis, chr; chromosome, clu; cluster, dPSI; delta percent spliced in, LD50; Lethal day 50%, LMN; lower motor neuron, PLS; primary lateral sclerosis, SALS, sporadic ALS, UMN; upper motor neuron.

Differential expression analysis identified 192 genes that were significantly altered (adjusted *P* < 0.05) between control and SALS donors (Fig. 2m and Supplementary Table 4). Hierarchical clustering of the differentially expressed genes revealed a strong segregation between control and SALS donors (Fig. 2n), with limited clustering of donors observed based on sex, disease classification, site of onset, age of onset, progression rate, donor survival or in vitro LD_50_ survival (Extended Data Fig. 6a). The most significantly changed genes were associated with functions related to autophagy, oxidative stress, cytokine production and RNA binding, while gene set enrichment analysis (GSEA) identified significant dysregulation across a large number of biological processes implicated in ALS. Prominent upregulation of genes involved in RNA metabolism (RNA localization, transport and processing), nucleocytoplasmic transport (protein import, localization and transport to the nucleus), vesicle transport (Golgi and endoplasmic reticulum vesicle transport and organization) and DNA damage repair (DNA repair and recombinational repair) was identified in SALS donors (Fig. 2o and Supplementary Table 5). Evidence of dysfunction in protein homeostasis, mitochondria and axons/synapses was also observed in the enriched cellular component and molecular function categories (Extended Data Fig. 6b,c and Supplementary Table 5). The identification of significant differential expression in patient-derived SALS motor neurons represents a major advancement for ALS, providing important insights into key pathogenic mechanisms conserved in SALS motor neurons.

Splicing analysis additionally identified 586 differentially expressed clusters, including 436 clusters containing cryptic exons, supporting the presence of dysfunctional splicing in SALS motor neurons (Supplementary Table 6). The differentially expressed clusters included mis-splicing and cryptic exon expression in *UNC13A*, a trend in *STMN2* (adjusted *P* = 0.0510), and overlap with transcripts associated with TDP-43 loss of function^29^ (Extended Data Fig. 6d,e and Supplementary Tables 7 and 8). Interestingly, the mis-splicing and cryptic exon expression in *UNC13A* and *STMN2* differ from the sites previously reported due to TDP-43 depletion or in the cortical neurons of patients with ALS^29–31^.

Finally, the expression of SALS differentially expressed genes identified in vitro (adjusted *P* < 0.05; Fig. 2s) was compared to existing transcriptional profiling of 52 postmortem spinal cords published by Humphrey et al.^32^ and used in previous iPS cell comparisons^25^. Comparison of the in vitro SALS disease profile and postmortem spinal cord gene expression profile generated a significant correlation for the cervical (*P* < 0.0001, *r* = 0.53) and thoracic (*P* < 0.0001, *r* = 0.71) spinal cords (Fig. 2p–r). A lower correlation was detected for the lumbar spinal cord (Fig. 2p–s; *P* = 0.003, *r* = 0.21), consistent with the limited expression of this motor neuron population in the cultures (Extended Data Fig. 6f). No significant correlation was detected when the order of the test-set genes was randomized (Extended Data Fig. 6g–i). These results support the idea that motor neurons derived from patients with SALS can recapitulate the pathophysiological processes occurring in the motor neurons of patients.

### Pharmacological attenuation of ALS phenotypes with riluzole

**Fig. 3:**
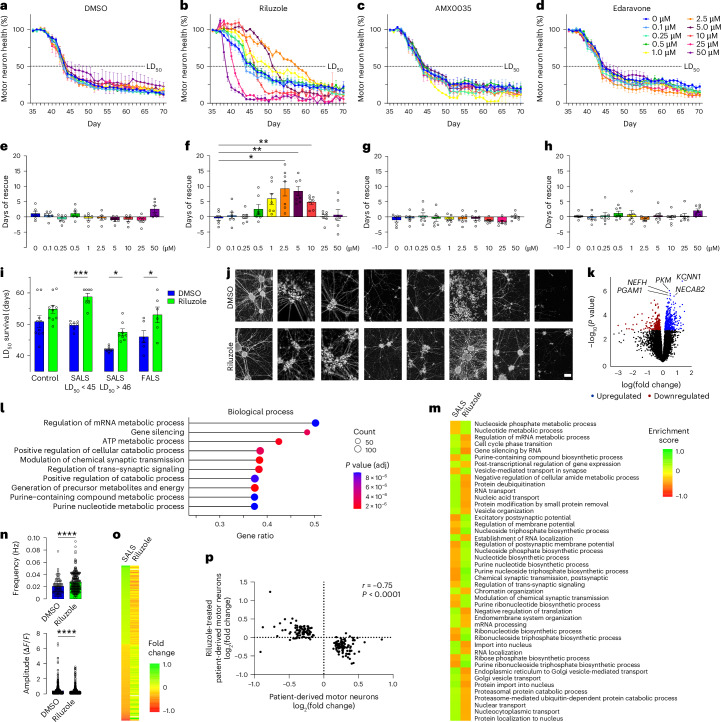
Pharmacological rescue of motor neuron health in patient-derived motor neurons. **a**–**d**, Longitudinal quantification of the total neurite length of HB9-tGFP^+^ motor neurons for a representative SALS donor treated with DMSO control (**a**) or FDA-approved disease-modifying treatments for ALS (**b**–**d**) (0–50 µM, expressed as a percentage of the pretreatment (day 36) value, mean of four wells/technical replicates ± s.e.m.). **e**–**h**, Quantification of the days of rescue (treated LD_50_ − DMSO LD_50_) of motor neurons from seven SALS donors (LD_50_ < 47) treated with DMSO control (**e**) or FDA-approved disease-modifying treatments for ALS (**f**–**h**) (0–50 µM, mean ± s.e.m., Brown–Forsythe ANOVA test, riluzole main effect *P* < 0.0001, 2.5 µM riluzole *P* = 0.045, 5 µM riluzole *P* = 0.005, riluzole 10 µM *P* = 0.004, *n* = 7 donors). **i**, Quantification of LD_50_ survival in motor neurons treated with 2.5 µM riluzole (riluzole treatment *P* < 0.0001, ALS status *P* < 0.0001, interaction *P* = 0.326) from healthy control (*P* = 0.126), SALS (LD_50_ < 45, *P* = 0.037), SALS (LD_50_ > 46, *P* = 0.0004) and FALS (*P* = 0.017) donors (mean ± s.e.m., two-way ANOVA with Šídák’s multiple comparisons test, *n* = 10 control donors, *n* = 8 SALS (LD_50_ < 45) donors, *n* = 7 SALS (LD_50_ > 46) donors, *n* = 6 FALS donors). **j**, Representative live-cell images (day 46) of motor neurons from eight SALS donors (LD_50_ < 45) treated with 2.5 µM DMSO control (top) or riluzole (bottom). **k**, Gene expression (RNAseq) volcano plot of ALS donors treated with DMSO control or riluzole, representing fold changes and *P* values; 596 genes were identified as differentially expressed (adjusted *P* < 0.05 highlighted, five most significant genes labeled, empirical Bayes moderated *t* statistics (limma–voom) with Benjamini–Hochberg multiple testing adjustment, *n* = 9 donors per group). **l**, Top 10 significantly overrepresented Gene Ontology gene sets (GSEA) for biological processes identified between ALS donors treated with DMSO control or riluzole (adjusted *P* < 0.01, Kolmogorov–Smirnov test with Benjamini–Hochberg multiple testing adjustment). **m**, Heatmap of GSEA enrichment scores for biological process Gene Ontology gene sets identified in SALS donors and the corresponding enrichment in riluzole-treated ALS donors. **n**, Average frequency (*P* < 0.0001) and amplitude (*P* < 0.0001) of calcium transients from untreated and riluzole-treated ALS motor neurons stained with Rhod-3 calcium-sensing dye (mean ± s.e.m., two-sided Mann–Whitney *U* test, DMSO frequency *n* = 189 cells/technical replicates and riluzole frequency *n* = 329 cells/technical replicates, DMSO amplitude *n* = 1,496 events and riluzole amplitude *n* = 2,976 events). **o**,**p**, Heatmap (**o**) and Spearman’s correlation analysis (**p**) of 192 differentially expressed genes in SALS motor neurons compared to riluzole-treated ALS motor neurons (two-sided). *P < 0.05, **P < 0.01, ***P < 0.001, ****P < 0.0001. Scale bar (**j**), 100 µm. Hz, hertz; *F*, fluorescence intensity.

**Extended Data Fig. 7:**
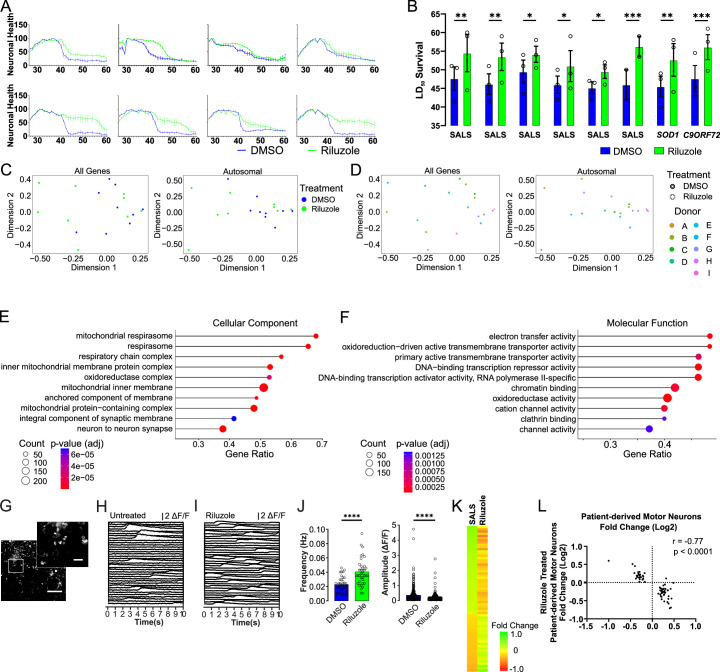
Pharmacological rescue, transcriptional profiling and calcium imaging of riluzole treated SALS motor neurons. **a** Longitudinal quantification of total Hb9-tGFP^+^ motor neuron neurite length in a subset of SALS donors (LD50<46) treated with DMSO or riluzole (expressed as percentage of peak neurite length, mean ± SE, n=4 wells/technical replicates). **b** Quantification of LD_50_ survival in motor neurons from ALS donors treated with 2.5 µM riluzole treatment (mean ± SEM, 2-way RM ANOVA with uncorrected Fisher’s LSD, riluzole treatment P<0.0001, subject P<0.0001, SALS P=0.004, SALS P=0.002, SALS P=0.034, SALS P=0.024, SALS P=0.047, SALS P=0.0007, SOD1 P=0.002, C9ORF72 P=0.0006, n=3 independent experiments). **c-d** Principal components plots (PC1-PC2) of RNAseq profiling for all genes (left) or autosomal genes only (right) in Riluzole and DMSO treated SALS motor neurons highlighting treatment group or individual donors (n=8 donors). **e-f** Top 10 significantly overrepresented gene ontology gene sets (GSEA) for cellular component and molecular function between Riluzole and DMSO treated SALS motor neurons (adjusted P value <0.01, Kolmogorov-Smirnov test with Benjamini–Hochberg multiple testing adjustment). **g** Image of ALS patient-derived motor neurons treated with the calcium indicator, GCaMP. Inset, magnified image of motor neurons. **h** Calcium transients from an example DMSO treated motor neuron indicated in (G). **i** Calcium transients from an example motor neuron treated with Riluzole. **j** Frequency (P<0.0001) and amplitude (P<0.0001) of the calcium transients in DMSO treated and riluzole treated motor neurons shown in (G) (mean ± SEM, Mann-Whitney U test, frequency n=36 cells/technical replicates, DMSO amplitude n=300 and riluzole amplitude n=537 events). **k** Heatmap and **l** Spearman’s correlation analysis of 192 differentially expressed genes in SALS motor neurons compared to riluzole treated SALS motor neurons (two-sided). Scale bar G, 500 µm, insert 100 µm. Abbreviations: adj; adjusted; DMSO, dimethyl sulfoxide; Hz; hertz; F, fluorescence intensity; LD50, Lethal day 50%; PLS, primary lateral sclerosis; SALS, sporadic ALS.

The need for a preclinical model of SALS remains a critical roadblock to the development of effective treatments for patients. To implement our system for preclinical drug testing, motor neurons were treated with three drugs recently approved for treating ALS (riluzole, edaravone and AMX0035). A comprehensive dose–response study of each drug (0.1–50 µM) was conducted on seven SALS donors (LD_50_ < 47) and assessed using longitudinal live-cell imaging (Fig. 3a–h). Riluzole treatment resulted in a significant rescue of LD_50_ survival at 2.5 µM (*P* = 0.045), 5 µM (*P* = 0.005) and 10 µM (*P* = 0.004), with a notable difference in efficacy between SALS donors (Fig. 3f). No significant effect of edaravone or AMX0035 was detected at any concentration (Fig. 3g,h), consistent with recent real-world and late-stage clinical trial data, respectively. The cell-autonomous rescue of patient-derived motor neurons by riluzole was then tested at 2.5 µM in healthy control donors, SALS donors with a strong survival deficit, SALS donors with a mild phenotype and FALS donors (Fig. 3i,j and Extended Data Fig. 7a). LD_50_ survival showed a significant interaction with donor (*P* < 0.0001) and treatment effect (*P* < 0.0001). Riluzole treatment resulted in a significant increase in the LD_50_ survival of motor neurons from SALS donors with a strong survival deficit (LD_50_ < 45, *P* = 0.037), SALS donors with a mild survival deficit (LD_50_ > 46, *P* = 0.0004) and FALS donors (*P* = 0.017). No significant rescue of LD_50_ survival was observed in the control donors, although at least some control donors appeared to be responsive to treatment. Replication of the findings across biological replicates (*n* = 3 independent differentiations) identified a significant extension in the LD_50_ survival of motor neurons for eight donors tested following riluzole treatment (Extended Data Fig. 7b).

To support the finding that riluzole treatment reversed the disease process in patient-derived motor neurons, transcriptional profiling was conducted following riluzole treatment (Extended Data Fig. 7c,d). RNAseq profiling of riluzole-treated motor neurons from nine SALS donors identified 593 differentially expressed genes compared to treatment with dimethyl sulfoxide (DMSO) control (Fig. 3k and Supplementary Table 9). Riluzole treatment resulted in the downregulation of genes associated with energy metabolism, glucose metabolism and synaptic function (Fig. 3l), revealing insights into the cell-autonomous mechanisms of action of riluzole. The findings were reinforced by enriched cellular component categories associated with the mitochondrial respirasome, glutamate receptors and synaptic signaling, along with related molecular function categories (Extended Data Fig. 7e,f and Supplementary Table 10). Compared to untreated SALS motor neurons (Fig. 2q), 45 of the enriched biological process Gene Ontology categories were reversed by treatment with riluzole (Fig. 3m and Supplementary Table 11), suggesting that the treatment alleviated the ALS disease processes associated with RNA and energy metabolism. Riluzole treatment also significantly increased the average calcium transient frequency (*P* < 0.0001) and significantly decreased the average amplitude (*P* < 0.0001) of ALS motor neurons when assessed using calcium imaging (Fig. 3n and Extended Data Fig. 7g–j). Enhanced spontaneous calcium activity in patient-derived ALS motor neurons supported the preservation of function induced by riluzole. Finally, a direct comparison of the SALS (192 differentially expressed genes) and riluzole-treated gene expression levels identified a significant negative correlation (*P* < 0.0001, *r* = −0.75; Fig. 3o,p), indicating a reversal of the SALS disease profile. This correlation increased when the profiled genes were restricted to those that matched the direction of the postmortem spinal cords, thereby increasing confidence in their relevance to the disease (*P* < 0.0001, *r* = −0.77; Extended Data Fig. 7k,l). Thus, we reproduced the efficacy of riluzole in SALS iPS cell-derived motor neurons, establishing a critical benchmark for future SALS preclinical testing.

### Screening of clinically tested ALS drugs using patient-derived SALS motor neurons

**Fig. 4:**
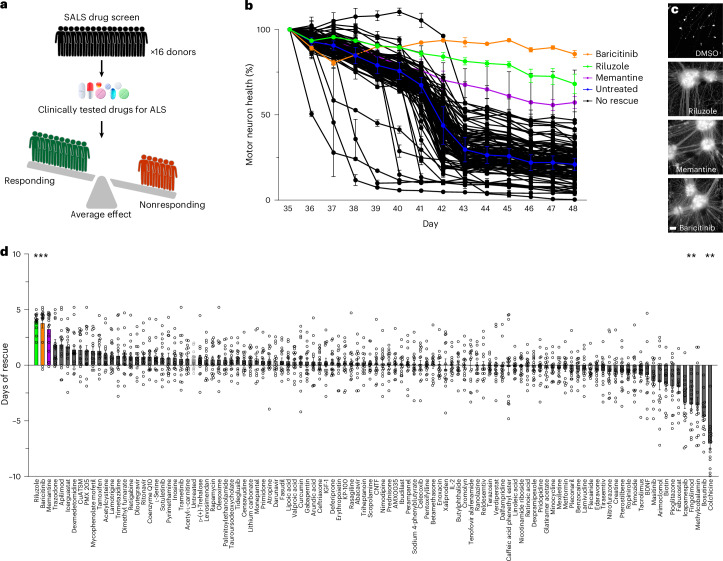
Reassessment of clinically tested drugs for ALS in motor neurons from SALS donors. **a**, Schematic of the drug screen with 107 drugs previously tested in ALS clinical trials using patient-derived motor neurons from ALS donors across the patient population. **b**, Longitudinal quantification of the total neurite length of HB9-tGFP^+^ motor neurons following treatment with clinically tested drugs for ALS in a representative SALS donor (2.5 µM, expressed as a percentage of the pretreatment (day 36) value, mean of two wells/technical replicates ± s.e.m.). **c**, Images of motor neuron health at the screening endpoint (HB9-tGFP^+^ motor neuron clusters and neurites at 20×). **d**, Quantification of the average days of rescue of motor neurons from 16 SALS donors (one dot per donor, mean ± s.e.m., Kruskal–Wallis statistic with Dunn’s multiple comparisons test, main effect *P* < 0.0001, riluzole *P* = 0.0003, baricitinib *P* = 0.0049, memantine *P* = 0.0066, icapamespib *P* = 0.0135, fingolimod *P* = 0.0078, bosutinib *P* = 0.009 and colchicine *P* = 0.003, *n* = 16 donors). *P < 0.05. Scale bar (**c**), 100 µm.

In a first for ALS drug screening, we then sought to conduct a primary drug screen on motor neurons derived from patients with SALS. To capture the heterogeneity in the SALS patient population, drug efficacy was screened on motor neurons derived from 16 SALS donors (Fig. 4a). The screening aimed to reassess the efficacy of all drugs that have previously undergone evaluation in phase 1–3 clinical trials as disease-modifying treatments for ALS. Using clinical trial databases, 169 interventions aimed at modifying disease progression were identified in ALS trials (excluding nutritional and biological therapies). A total of 107 drugs compatible with drug screening and commercially available were sourced, and their efficacy at a concentration of 2.5 µM was assessed on motor neurons from SALS donors with established survival deficits (LD_50_ < 47). A majority of the drugs tested (black lines) did not improve motor neuron health, with only three drugs—riluzole, baricitinib and memantine—showing evidence of efficacy over the time course (Fig. 4b,c). Drug efficacy across the SALS population was assessed by quantifying the change in LD_50_ survival relative to DMSO treatment (days of rescue) for each drug (Fig. 4d (each dot represents an individual donor, *P* < 0.0001) and Supplementary Table 12). Riluzole (*P* = 0.0003), baricitinib (*P* < 0.0049) and memantine (*P* = 0.0066) produced a significant rescue of motor neuron health, noting that the length of the screen limited the maximum detectable rescue. Additionally, colchicine, bosutinib, fingolimod and icapamespib treatment significantly reduced LD_50_ survival in the screen. It is important to note that the drug concentrations used in the screen may differ from those used clinically, and individual results from the drug screen must be validated before drawing conclusions about drug effects in a clinical setting. The scale of the drug screen implemented in this study represents a moderately sized screen (107 drugs tested in duplicate wells across 16 donors); however, the scale is limited only by the practicalities and costs associated with iPS cell differentiation and the length of the assay. The capability to rapidly assess drug efficacy and toxicity across the SALS patient population provides an important tool for ALS drug development.

### Preclinical assessment of riluzole, memantine and baricitinib in patient-derived motor neurons

**Fig. 5:**
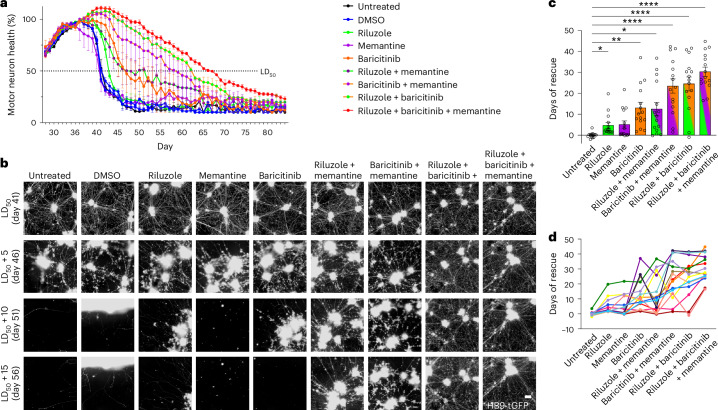
Preclinical testing of baricitinib, memantine and riluzole in motor neurons from SALS donors. **a**, Longitudinal quantification of the total neurite length of HB9-tGFP^+^ motor neurons in a representative SALS donor treated with riluzole, memantine and baricitinib as monotherapies or combinations (total neurite length of HB9-tGFP^+^ motor neurons, mean of four wells/technical replicates ± s.e.m.). **b**, Images of the motor neurons from **a** at the screening endpoint (HB9-tGFP^+^ motor neuron clusters and neurites at 20×). **c**, Quantification of the average days of rescue of motor neurons from 15 SALS donors after treatment with riluzole, baricitinib and memantine as monotherapies or combinations (one dot per donor, mean ± s.e.m., Brown–Forsythe ANOVA with Dunnett’s T3 multiple comparisons test, main effect *P* < 0.0001, riluzole *P* = 0.0257, memantine *P* = 0.0739, baricitinib *P* = 0.0014, riluzole + memantine *P* = 0.0109, baricitinib + memantine *P* < 0.0001, riluzole + baricitinib *P* < 0.0001, riluzole + baricitinib + memantine *P* < 0.0001, *n* = 15 donors). **d**, Days of rescue from **c** represented per individual (one line per donor, *n* = 15 donors). *P < 0.05, **P < 0.01, ****P < 0.0001. Scale bar (**b**), 100 µm.

**Extended Data Fig. 8:**
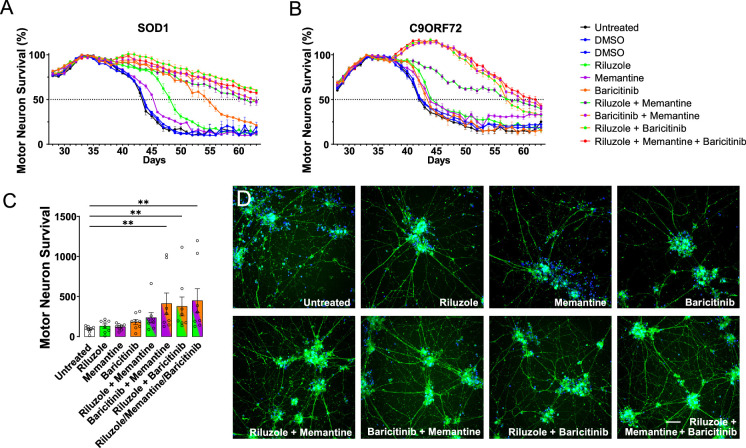
Pre-clinical testing of baricitinib, memantine and riluzole in sporadic and familial ALS donor motor neurons. **a-b** Longitudinal quantification of total Hb9-tGFP+ motor neuron neurite length in familial ALS (FALS) donors harbouring a **a**) SOD1I114T variant or **b**) C9ORF72 G4C2>145x expansion treated with riluzole, baricitinib and memantine as monotherapies or combinations (total Hb9-tGFP+ motor neuron neurite length, mean ± SEM, n=4 wells/technical replicates). **c** Quantification of ChAT immunoreactive cell number at Day 49 in sporadic ALS donors treated with riluzole, baricitinib and memantine as monotherapies or combinations (one dot per donor, mean ± SEM, Brown-Forsythe ANOVA with Dunnett’s T3 multiple comparisons test, main effect P=0.010, baricitinib/memantine P=0.006, baricitinib/riluzole P=0.024, riluzole/memantine/baricitinib P=0.003, n=9 donors). **d** Images of motor neuron health at screen endpoint (Hb9-tGFP+ motor neuron clusters and neurites at 20x). Scale bar D, 100µm Abbreviations: DMSO; dimethyl sulfoxide.

To validate the therapeutic potential of baricitinib and memantine on SALS motor neurons, preclinical testing with a prolonged time course was conducted on 15 SALS donors. Riluzole, baricitinib and memantine were tested as monotherapies, in two-drug combinations or as a three-drug combination at 2.5 µM (Fig. 5a,b). Quantification of LD_50_ survival for the SALS population (Fig. 5c, each dot represents an individual donor, *P* < 0.0001) resulted in a significant rescue following treatment with riluzole (+4.7 days, *P* = 0.0257) and baricitinib (+13 days, *P* = 0.0014)—but not memantine—as monotherapies. In combination, treatment with memantine and riluzole (+12.5 days, *P* = 0.0109); baricitinib and memantine (+23.5 days, *P* < 0.0001); baricitinib and riluzole (+24.5 days, *P* < 0.0001); or riluzole, memantine and baricitinib (+30.3 days, *P* < 0.0001) significantly increased the LD_50_ survival of the SALS population. Assessing the response of individual donors within the treated donor population, the combinations of memantine + baricitinib and riluzole + baricitinib resulted in a strong rescue in 87% of the donors (>10 days). Notably, the number of responding donors increased to 100% following treatment with riluzole, memantine and baricitinib, with the minimum rescue duration for an individual donor increasing to more than 16 days. This result indicates that the triple-drug combination may not only increase the average benefit for the SALS population but also provide benefits to donors who failed to respond to other combinations (Fig. 5d). Rescue of motor neuron health was also observed in FALS donors harboring variants in *SOD1* or repeat expansions in *C9ORF72* (Extended Data Fig. 8a,b). Quantification of ChAT-immunolabeled cultures at day 49 confirmed a significant increase in the survival of SALS motor neurons following treatment with baricitinib and memantine (*P* = 0.006); baricitinib and riluzole (*P* = 0.024); or riluzole, memantine and baricitinib (*P* = 0.003; Extended Data Fig. 8c,d). The successful identification and preclinical validation of three-drug combinations using patient-derived SALS motor neurons demonstrate the power of iPS cell technology to measure drug efficacy across the SALS patient population and represent an important milestone in drug development for ALS.

## Discussion

Patient-derived iPS cells have enormous potential to transform our understanding and treatment of heterogeneous and complex neurodegenerative diseases such as ALS, Alzheimer’s disease and Parkinson’s disease. While advances have been made in modeling rare familial and monogenic forms of neurodegenerative diseases, difficulties in replicating key pathologies in neurons derived from patients with sporadic disease, which constitute 80–90% of the disease burden, have greatly hindered progress. Despite intensive investment in the generation of iPS cell libraries of unprecedented scale^23^, along with deep omics analysis of differentiated neurons, little convincing evidence has emerged showing that sporadic disease can be modeled with the technology.

In this study, we generated a highly curated ALS iPS cell library complemented by clinical profiling and genetic characterization of the donors. Including more than 100 ALS donors, the library aimed to capture the full spectrum of ALS clinical and biological subtypes in the patient population and reflects the scale of a well-powered mid-phase clinical trial. Acknowledging the importance of replicating a genuine neurodegenerative phenotype, extensive investments were made in the development of a robust motor neuron differentiation and phenotyping protocol capable of quantifying variable and subtle phenotypes in patients with SALS. Terminal differentiation of iPS cells was conducted using small molecules and culture conditions that mimic those in the human spinal cord, avoiding the use of artificial induction or integrating vectors. By implementing a high-stringency confirmation of a spinal motor neuron phenotype (triple positive for Tuj1, HB9 and ChAT), the protocol generated more than 90% pure motor neurons. Coupled with automated longitudinal live-cell phenotypic screening to capture the spontaneous, rapid and heterogeneous degeneration of motor neurons exhibited across the iPS cell library, along with an intuitive and versatile measure of motor neuron health (LD_50_ survival), the system provided a sensitive and reproducible model of motor neuron survival for phenotyping and therapeutic testing.

Large-scale phenotypic assessment revealed a significant reduction in the survival of motor neurons and neurites (LD_50_ survival) from patients with SALS, recapitulating a key pathological hallmark of ALS. With a sufficient number of donors to provide statistical power, the findings represent convincing evidence that patient-derived SALS motor neurons undergo spontaneous accelerated degeneration in vitro. A significant correlation between the patient-derived motor neuron phenotype (LD_50_ survival) and the donors’ clinical survival time also provided a direct link between the modeled phenotype and the clinical aspects of individual patients in real-life settings. Transcriptional profiling successfully identified significant differential gene expression between SALS and control motor neurons, a finding that is highly notable, as large-scale profiling of patient-derived motor neurons has previously failed to detect significant differences in patient-derived SALS motor neurons^23,25^. Converging dysfunctions in RNA metabolism, nucleocytoplasmic transport, endoplasmic reticulum–Golgi vesicle transport and DNA damage repair were identified as mechanisms driving dysfunction in SALS. These disease pathways are highly consistent with the postulated ALS disease mechanisms identified from causal genetic variants in monogenic ALS^9^, providing valuable insights into the mechanisms driving neurodegeneration. The SALS disease profile of patient-derived motor neurons also correlated significantly with the transcriptional profiles of postmortem spinal cords, providing evidence that the disease phenotype in cultured motor neurons replicates the disease process occurring in patients.

The clinical relevance of the model was supported by the pharmacological rescue of impaired LD_50_ survival with riluzole. Riluzole remains the only drug unambiguously supported by clinical trials to slow ALS progression^1–3^; however, rescue of motor neurons has not previously been demonstrated in iPS cells^15^ or consistently in animal models^33^. Supported by transcriptional profiling and calcium imaging that show a reversal of the disease process, evidence of a cell-autonomous mechanism of action for riluzole directly on motor neurons (in the absence of astrocytes) provides insight into how and when the drug may confer therapeutic benefit clinically^34–36^. The utility of the model for drug testing in SALS was further emphasized by the failure to identify significant efficacy for edaravone or AMX0035. Regulatory approval of these treatments for ALS was controversial, as limited clinical evidence or early-stage trial data were available to support their efficacy^37,38^. Despite mechanisms of action purportedly targeting motor neurons, the findings of this study do not support their efficacy on motor neurons derived from patients with SALS. These results are consistent with recent clinical trial data for both drugs^39^.

The cumulative evidence of impaired survival, clinical correlations, transcriptional profile and pharmacological rescue in patient-derived motor neurons described here provides important face validity that a pathophysiologically relevant SALS model has been generated. The replication of a cell-autonomous survival deficit encompassing the complex disease processes driving SALS is an important advance over existing drug screens, preclinical models^14,19–22,40^ and monogenic patient-derived models^15,18,24^, which is likely to increase the chance of identifying drugs that will translate into effective treatments in clinical trials. However, the approach does not represent a perfect model of SALS. While the highly pure cultures generated provide a powerful reductionist system for modeling motor neurons, the important contributions of upper motor neurons and other non-neuronal cell types, such as astrocytes and microglia, are not captured. Biological and technical variability in the LD_50_ survival of motor neurons was also observed. It is likely that evolving screening methods and advances in iPS cell development^41^ will continue to provide more accurate and sensitive modeling of SALS, building on the approach described here. Additionally, TDP-43 nuclear depletion or cytoplasmic mislocalization was not identified despite evidence of broad splicing changes and cryptic exon inclusion in patient-derived SALS motor neurons that are consistent with TDP-43 dysfunction. It is feasible that higher-sensitivity or single-cell resolution technologies may be required to reveal evidence of TDP-43 pathologies across the complex cellular and patient populations examined, or that additional stressors or cell types are needed. It is also feasible that SALS motor neuron degeneration does not require TDP-43 mislocalization, favoring the involvement of TDP-43 dysfunction or partial loss of function. ALS-like symptoms and motor neuron loss occur without TDP-43 pathology in the majority of TDP-43 mouse models^42–47^, placing pathology such as cytoplasmic TDP-43 mislocalization and aggregation as a potential downstream disease process. Concordance between our results, other SALS iPS cell models^25^ and mouse models highlights the ongoing debate in the field regarding the role of TDP-43 mislocalization and pathology in SALS.

The implementation of drug testing across the SALS patient population in this study (15+ donors) establishes an important benchmark for drug screening in ALS. The failure of 97% of the drugs that have undergone clinical trials for ALS to show efficacy on SALS motor neurons in vitro closely mirrors the clinical trial outcomes for these drugs and contrasts strongly with the preclinical testing of the drugs using established animal models. These results suggest that motor neurons derived from patients with SALS may provide improved stringency and clinical translatability for ALS drug development and preclinical testing. Only baricitinib, riluzole and memantine significantly increased motor neuron health in the screening. Baricitinib is a JAK1/2 inhibitor, and blocking the JAK–STAT signaling pathway has been demonstrated to reverse the innate immune response induced by double-stranded RNA in models of ALS^48^. It was also identified as a key target to reduce ALS pathophysiology in neurons, glia, muscle fibers and blood cells, using the BenevolentAI knowledge graph^49^. Memantine is an *N*-methyl-d-aspartate receptor antagonist that targets excitotoxicity^50^, with conflicting evidence from initial clinical trials^51–53^. It has recently undergone testing in two phase 2/3 clinical trials (MND-SMART (NCT04302870) and TAME (NCT02118727)).

Preclinical validation of the drugs to model LD_50_ survival in patient-derived SALS motor neurons confirmed a significant rescue of LD_50_ survival following treatment with baricitinib (as a monotherapy). This result provides a third independent line of evidence supporting the use of baricitinib as a therapeutic for ALS, making the drug a highly promising candidate for clinical trials. In contrast, memantine did not show a significant survival benefit—a result that aligns with the two recent phase 2/3 clinical trials, MND-SMART and TAME, where memantine failed to demonstrate significant efficacy in patients^54^.

Combinatorial testing of two- and three-drug combinations of riluzole, memantine and baricitinib identified significantly increased efficacy for all combinations. The LD_50_ rescue of the two-drug combination of baricitinib and memantine was 5.0 times that of riluzole alone, while the combination of baricitinib and riluzole produced an LD_50_ rescue 5.2 times that of riluzole alone. Both treatments resulted in 87% of donors responding to the treatment, with a minimum benefit of 10 days of rescue. The three-drug combination of riluzole, memantine and baricitinib increased the average rescue to 6.5 times that of riluzole alone, with 100% of donors responding to the drug combination (minimum rescue of 16.5 days). With each drug acting through different mechanisms, the synergistic benefits afforded by combining the treatments strongly support the use of combinatorial therapies in ALS and other complex neurodegenerative diseases. The estimation of the proportion of patients who may benefit from a treatment is an important advance that may allow for improved selection of drug candidates for clinical trials and better planning of those trials. Population-level screening may also enable the investigation of responding and nonresponding donors to develop molecular or genetic biomarkers that inform patient selection in clinical trials.

This study sets several benchmarks for disease modeling and drug testing in ALS and other neurodegenerative diseases. The recapitulation of a well-validated disease phenotype in motor neurons from SALS donors greatly enhances drug discovery and preclinical testing capabilities for ALS, which is predominantly sporadic, heterogeneous and idiopathic. The successful screening and preclinical testing of drug efficacy across the SALS population represent a major milestone for drug discovery in ALS and removes a major roadblock for therapeutic development. The potential of the approach is exemplified by the rapid identification and preclinical testing of baricitinib-based drug combinations, which afford rescue up to 6.5 times that of the current standard of care in ALS. The drug combinations are particularly attractive in light of the high rescue rates and the number of responding donors identified during testing. Baricitinib and memantine are reported to be CNS (central nervous system) penetrant; are approved by the US Food and Drug Administration (FDA), European Medicines Agency and Therapeutic Goods Administration of Australia; and have well-established safety profiles, enabling the rapid delivery of the combination to clinical trials.

## Methods

### Ethics

The collection of human tissues, clinical data and demographic information complied with all relevant ethical regulations and was approved by the University of Melbourne Human Research Ethics Committee (ID 1749960). Informed consent was obtained from all participants through a standardized and interactive multimedia consent process^55^, permitting the use of the iPS cell lines and data for motor neuron disease/ALS and related research. Participants did not receive compensation for their involvement in the study.

### Patient recruitment

Volunteers diagnosed with motor neuron diseases (including ALS, PLS, spinal and bulbar muscular atrophy, and adult-onset spinal muscular atrophy) were recruited from all states in Australia. Demographic and clinical information was collected by the Australian Motor Neuron Disease Register and the SALS Australian Systems Genomics Consortium. Skin biopsy specimens and blood samples from patients were collected between December 2017 and August 2018. Volunteers with no history of neurological diseases and an average age beyond the typical onset of ALS (to reduce the risk of neurological comorbidities) were recruited from latitude-matched locations to act as healthy controls.

### Whole-genome sequencing

DNA was extracted from whole blood using a DNeasy Blood and Tissue Kit (Qiagen) including on-column RNase treatment, following the manufacturer’s instructions. Whole-genome sequencing libraries were prepared using TruSeq DNA PCR-free library preparation kits (Illumina) and sequenced on a NovaSeq 6000 sequencer (Illumina) to approximately 30× coverage with 150-bp single-end reads. Variant identification was conducted as described in the Supplementary Note, following best-practice workflows for germline short variant discovery in GATK (Genome Analysis Toolkit)^56^.

#### Structural variant calling

ExpansionHunter (v4) was used to call repeat variants in *C9ORF72* and *AR* (Kennedy’s disease, also known as spinal and bulbar muscular atrophy). The repeat size thresholds for calling structural variants from ExpansionHunter outputs were as follows: *C9ORF72*, risk > 23–29 repeats, pathogenic > 29 repeats^57,58^; *AR*, pathogenic > 37 repeats^59,60^. *C9ORF72* expansions were confirmed with repeat primed PCR using an AmplideX PCR/CE C9orf72 Kit. Genomic DNA was PCR amplified using a three-primer G4C2-Repeat Primed PCR following the manufacturer’s instructions and quantified using fragment analysis on a genetic analyzer (Applied Biosystems). *AR* repeat lengths were confirmed as described previously^61^ and quantified using fragment analysis on a genetic analyzer (Applied Biosystems).

### iPS cell reprogramming

Primary fibroblasts were isolated from skin biopsy specimens and reprogrammed using nonintegrating vectors in feeder-free conditions^62^ with automated cell handling robotics (Tecan) to minimize variation, as previously described^26^. Skin punch biopsy samples (3–4 mm) were collected in DMEM with 100 U ml^−1^ penicillin, 100 μg ml^−1^ streptomycin and 250 ng l^−1^ fungizone (Life Technologies). The tissue was treated with 1.8 U ml^−1^ dispase in HBSS (Life Technologies) at 4 °C overnight; the epidermis and subcutaneous fat were removed; and the dermis was cut into small pieces. The dermis pieces were cultured at 37 °C and 5% CO_2_ in DMEM supplemented with 10% FBS (Sigma), 2 mM GlutaMAX, 100 U ml^−1^ penicillin and 100 μg ml^−1^ streptomycin (Life Technologies). Fibroblasts were passaged using 0.05% trypsin–EDTA (Life Technologies) at 60–80% confluency and frozen for reprogramming at passage 2.

Cryopreserved fibroblasts (0.5 × 10^6^ cells) were thawed and reprogrammed in batches of 20–60 using an automated platform (Tecan) under feeder-free conditions^26^. Fibroblasts were cultured on plates coated with Vitronectin XF (Stem Cell Technologies) using the medium described above. Reprogramming was conducted by episomal nucleofection using a Basic Fibroblast Nucleofector Kit (Lonza) with OCT4, SOX2, KLF4, L-MYC and LIN28 vectors and p53-targeting shRNA^62^. Nucleofected fibroblasts were cultured in E7 medium (Stem Cell Technologies), and pluripotent stem cells were selected by magnetic sorting using anti-human TRA-1-60 microbeads (Miltenyi Biotec). Reprogrammed iPS cells were cultured in StemFlex (Life Technologies) and passaged using ReLeSR (Stem Cell Technologies) weekly for 8 weeks, confirming mycoplasma negativity with a MycoAlert Mycoplasma Detection Kit (Lonza)^63^. Reprogramming and karyotypic analysis for a single donor (ID 797, harboring a *UBQLN2* variant) were performed separately using peripheral blood mononuclear cells isolated with a BD Vacutainer CPT (BD Biosciences) and reprogrammed using a Cytotune-iPS 2.0 Sendai Reprogramming Kit, as previously described^64^.

### iPS cell quality control

Assessment of karyotypic integrity, markers of pluripotency and trilineage germ layer differentiation potential was conducted using methods adapted from previous work^27,65^. DNA was extracted from donor blood and passage 8–10 iPS cells using a QIAamp DNA Mini Kit (Qiagen). Cell line authentication and virtual karyotyping were conducted using pairwise comparisons of donor iPS cells and blood to confirm the identity of iPS cell lines and exclude lines exhibiting genetic contamination. Samples were genotyped using the Illumina Infinium Global Screening Array-24 v2.0 BeadChip with multidisease drop-in (GSAMD-24v2-0_20024620_A4, Illumina) and analyzed as described in the Supplementary Note.

The pluripotency and trilineage potential of all iPS cells were assessed using gene expression profiling of passage 8–10 iPS cells and differentiated embryoid bodies (EBs) containing three lineages^65^. EBs containing three cell lineages were cultured in Essential 6 Medium (Life Technologies) for 16 days. RNA was isolated from independent iPS cell passages and EB differentiations (*n* = 3) using the High Pure RNA Isolation Kit (Roche); cDNA was reverse-transcribed using iScript RT Supermix (Bio-Rad); and qPCR was performed with an SsoAdvanced Universal SYBR Green Supermix (Bio-Rad). To provide a high-throughput approach for confirming pluripotency and trilineage potential, 78 genes derived from the qPCR ScoreCard assay—including pluripotency, ectoderm, endoderm and mesoderm markers, as well as housekeeping genes—were profiled on the WA09 (H9, WiCell) human embryonic stem cell line^66^ (primers listed in Supplementary Table 13). A reduced subset of gene expression markers providing a robust and stable gene expression signature specific to the medium conditions and protocols used to culture the cells was identified. The housekeeping gene (*RPS29*), pluripotency markers (*NANOG*, *LCK*, *IDO1*), ectoderm markers (*PAX6*, *PAX3*, *NR2F2*), endoderm markers (*SOX17*, *CPLX2*, *EOMES*) and mesoderm markers (*TBX3*, *FOXF1*, *HAND2*) were selected and used to assess pluripotency and trilineage potential for all iPS cell lines, including three H9 iPS cells and three EBs as positive and negative controls. Gene expression was quantified across all iPS cells using a D300e Digital Dispenser (Tecan) to minimize variation and was analyzed using the ΔΔCt method^67^ relative to expression in the H9 human stem cell line. Heatmaps to represent the data were generated in R using the gplots package^68^.

### Spinal motor neuron differentiation

#### Protocol development

Selection of an optimal spinal motor neuron differentiation protocol for phenotyping was determined by comparing leading and well-established differentiation protocols^23,28^. The protocol described in the study by Du et al.^28^ produced highly enriched motor neuron cultures; however, prolonged exposure to growth factors in stage 4 (standard protocol) led to the degeneration of motor neurons from both healthy controls and ALS donors on our platform during long-term culturing. An alternative protocol for drug screening described by Du et al.^28^ excluded the growth factors insulin-like growth factor-1 (IGF-1), ciliary neurotrophic factor (CNTF) and brain-derived neurotrophic factor (BDNF) in stage 4 (screening protocol), resulting in motor neurons that failed to fully thrive and mature on our platform. The resulting cultures exhibited high variability in motor neuron health between replicate experiments and were not pursued. Testing of the differentiation protocol implemented by Baxi et al.^23^ generated mixed cell populations with moderate numbers of motor neurons, which were deemed suboptimal for use on the platform due to the potential impact of variable non-motor neuron cell types on downstream phenotyping, transcriptional profiling and drug testing. To develop a robust protocol that generates consistent cultures of mature motor neurons across large numbers of iPS cell lines, comprehensive optimization was undertaken using FALS and control donors. Differentiation into spinal motor neurons was conducted using the differentiation protocol of Du et al.^28^ to take advantage of the highly pure motor neuron cultures generated. The protocol was modified to mimic the environment of the central nervous system (stages 1–5), optimize the health and maturation of the motor neurons (stage 4), and provide long-term culturing conditions for a disease phenotype to develop (stage 5).

#### Optimized protocol

The terminal differentiation to spinal motor neurons was conducted using small molecules, as previously described^28^ with the following modifications: cells were cultured on a human laminin-521 substrate (Biolamina) under hypoxic conditions (37 °C, 95% humidity, 5% O_2_ and CO_2_). On day 19, motor neuron progenitors were dissociated into a single-cell suspension using Accutase (Stem Cell Technologies) and seeded onto optically clear 384-well plates (Greiner). Motor neurons were matured in BrainPhys and NeuroCult SM1 Without Antioxidants (Thermo Fisher), containing 0.5 µM all-*trans* retinoic acid (Stem Cell Technologies), 0.1 µM purmorphamine (Stem Cell Technologies), 0.1 µM StemSelect Compound E (Calbiochem) and 20 ng ml^−1^ recombinant human IGF-1 (rhIGF-1), rhCNTF and rhBDNF (R&D Systems) until day 26 (stage 4). On day 25, cultures were treated with 5 µM cytosine β-d-arabinofuranoside (Ara-C) (Sigma-Aldrich) to prevent the proliferation of non-neuronal cells. From day 26, rhIGF-1, rhCNTF and rhBDNF were omitted from the medium. From day 32, purmorphamine was also omitted to avoid suppressing the disease phenotype (stage 5). A 50% medium change was conducted every 3–4 days for stages 4 and 5 of the protocol.

### Immunohistochemistry and cell quantification

Cultures were washed with PBS and fixed by immersion in 4% (wt/vol) paraformaldehyde for 2 h. Immunolabeling was performed overnight using the primary antibodies described in Supplementary Table 14, with the corresponding Alexa Fluor secondary antibodies applied for 2 h at room temperature at a dilution of 1:400 (Thermo Fisher Scientific). All antibodies were diluted in PBS containing 5% normal serum and 0.25% Triton X-100 (Amresco) and were incubated at room temperature. Z-stack images were captured using an Opera Phenix High-Content Screening System (PerkinElmer) at 20× magnification from four fields of view per well in four wells per donor.

Quantification of HB9, ChAT and Tuj1 triple-positive cells, Tuj1^+^ cells, GFAP^+^ cells and CD11B^+^ cells was conducted by manual counting. Cells were identified using DAPI staining of cell nuclei (excluding dead cells containing condensed chromatin) and immunolabeling for the respective cellular markers, which were individually assessed for each cell to assign cellular identity. Quantification of ChAT^+^ cell numbers and TDP-43 intensity was conducted using Harmony software (v5.1) with a customized RMS (Ready-Made Solution). Cell nuclei were identified using DAPI staining, and cytoplasmic delineation was conducted through ChAT immunolabeling (using method B for precise detection and a dual-border adjustment strategy of 3% outer and 45% inner for ChAT). Motor neurons were defined as cell nuclei surrounded by a ChAT-immunoreactive cytoplasm. The values of TDP-43 intensity within the nucleus and cytoplasmic regions of motor neurons were averaged for all cells per well.

### Quantification of neurite length

Motor neurons were labeled with an adenovirus expressing tGFP driven by a mouse *Hb9* enhancer fused to the mouse *Hsp68* minimal promoter (VectorBuilder) at a multiplicity of infection of 10 and delivered on day 21. Images of tGFP-labeled motor neurons were acquired every 24 h on a Cell Discoverer 7 (Zeiss) or Opera Phenix High-Content Screening System (PerkinElmer) live-cell imaging microscope at 20× magnification.

Images of motor neurons expressing tGFP were batch processed and analyzed using a custom preprocessing, segmentation and quantification pipeline developed in Knime (v4.6.3)^69^ (described in detail in the Supplementary Note). Total neurite length was calculated per image and averaged over three images per well. Wells with less than 12,500 μm total neurite length at all time points, failed neurite quantification in >25% of time points (excluding days 50–60, where failed quantification may result from degeneration) or an LD_50_ occurring before the maturation of the neurons (before day 39) were excluded from the analysis. To account for the differential growth characteristics between donors and simplify the visualization of the data, motor neuron neurite length was expressed as a percentage of the maximum neurite length across time points for each well (that is, plots displayed with peak neurite innervation standardized to 100% for each well). The neurite rate of growth was calculated as the maximum percentage increase over 5 days, while the neurite rate of decline was determined as the maximum percentage decrease over 2 days in each well. LD_50_ was defined as the day when neurite innervation dropped to 50% of its peak value in each well, or as the experimental endpoint if >50% was not reached.

### Phenotypic screening

Phenotypic screening of FALS and SALS donors was conducted using patient-derived motor neurons cultured in 384-well plates. Protocol validation was conducted on five control donors (averaged) and five FALS donors harboring variants or expansions in *SOD1*^*I114T*^, *TDP43*^*S393L*^, *C9ORF72* G_4_C_2_^>145×^, *VCP*^*R114C*^ and *UBQLN2*^*T487I*^ in three independent biological replicates. FALS replication was conducted on five donors harboring variants or expansions in *SOD1* (*SOD1*^*G94V*^, *SOD1*^*I114T*^, *SOD1*^*L145S*^, *SOD1*^*A96V*^, *SOD1*^*A4V*^) or *C9ORF72* (*C9ORF72* G_4_C_2_^>145×^), including two external lines (CS29iALS-C9n1 and CS52iALS-C9n6). The *SOD1*^*A4V*^ mutant and isogenic control were kindly provided by K. Eggan (Harvard University, Cambridge, MA, USA)^24,70^, while the *SOD1*^*I114T*^ mutant and isogenic control were generated in-house. CRISPR–Cas9 gene correction of the I114T point mutation was conducted using a guide RNA–Cas9 ribonucleoprotein complex and a single-stranded oligodeoxynucleotide donor template with a Neon NxT electroporation system (Thermo Fisher Scientific) at 1,050 V, 30 ms and two pulses (guide RNA 5′-TCA GGA GAC CAT TGC ATC AT, single-stranded oligodeoxynucleotide 5′-ATG TGT CTA TTG AAG ATT CTG TGA TCT CAC TCT CAG GAG ACC ATT GCA TCA TTG GCC GCA CAC TGG TGG TAA GTT TTC ATA AAA GGA TAT GCA TAA AAC T). Quality control of the line was conducted as described for the iPS cell library. The CS29iALS-C9n1 and CS52iALS-C9n6 lines, along with isogenic controls, were obtained from Cedars-Sinai Medical Center’s David and Janet Polak Foundation Stem Cell Core Laboratory. The length of GFP-positive neurites was quantified using the average of four replicate wells per donor, with three images or fields of view per well.

Population-wide phenotypic screening was conducted for all SALS donors (98 ALS donors and 25 healthy controls), with a total of 65 SALS donors, 4 donors with suspected PLS and 22 control donors producing motor neuron cultures that passed quality control requirements in a minimum of three wells. The length of GFP-positive neurites was quantified using the average of six replicate wells per donor, with four images or fields of view per well. Clinical data were log-transformed before correlation analysis when a log-normal distribution was observed.

### Transcriptional profiling of patient-derived ALS motor neurons

Patient-derived motor neurons were differentiated from all donors in the iPS cell library plus two additional FALS donors harboring variants in *FUS*^*R524S*^ (CS37iALS-FUSn3) and *VCP*^*R155H*^ (CS8RHDiALS-n2) obtained from Cedars-Sinai Medical Center’s David and Janet Polak Foundation Stem Cell Core Laboratory. RNA was isolated from duplicate wells in 48-well plates on day 49 using an Arcturus PicoPure RNA Isolation Kit (Thermo Fisher Scientific), which included on-column DNase treatment. RNA libraries were prepared with the Stranded Total RNA with Ribo-Zero Plus Library Prep Kit (Illumina) and sequenced on a NovaSeq 6000 sequencer (Illumina) to generate approximately 60 million 150-bp paired-end reads per sample. The day 49 time point was selected to enable a comparison of patient-derived motor neurons to postmortem motor neurons, resulting in the complete degeneration of cultures from a large number of donors. A total of 88 samples passed all quality control criteria, with sample exclusions due to failed differentiations; insufficient RNA (<60 ng); DV200 (percentage of RNA fragments >200 nucleotides in length) < 30%; failed library preparation; cell populations identified as statistical outliers for *MNX1*/*HB9*, *CHAT*, *ISL1* or *VACHT* expression (ROUT, *Q* = 1%); or divergent survival curves (LD_50_ > 60).

RNAseq transcriptomic analysis was conducted in the R statistical computing environment (v4.2.2)^71^. Raw reads were aligned to the human genome build 38, and counts were summarized to genes using the Rsubread (v2.12.2) package^72,73^. Gene annotations were acquired using the biomaRt (v2.54.0) package, with gene biotypes limited to protein coding. Genes with a minimum of 2 counts per million in six samples were subjected to voom normalization using edgeR (v3.40.1), variance partition analysis with variancePartition (1.34.0) and differential expression testing using the limma (v3.54.0) package^74,75^. Analysis was conducted using a design matrix that incorporated disease type and sex. Principal component analysis and volcano plots were generated in limma. Differentially expressed genes were identified between control and patient-derived SALS motor neurons using adjusted *P* values of <0.05. Expression heatmap generation and unsupervised clustering were performed using the gplots (v3.1.3) package^68^. GSEA was conducted on Gene Ontology gene sets using clusterProfiler (v4.8.3) with a customized background set and an adjusted *P*-value cutoff of 0.01.

RNAseq splicing analysis was conducted, with exon–exon junctions extracted and annotated using RegTools (v1.0.0)^76^. Clustering and differential splicing were quantified using the LeafCutter (v0.2.9) package^77^. Analysis was conducted using a design matrix that incorporated disease type and sex, and differentially expressed clusters were identified between control and patient-derived SALS motor neurons using adjusted *P* values of <0.05.

Correlation analysis was conducted on log_2_(fold-change) RNAseq data from 192 differentially expressed genes (false discovery rate < 0.5) identified between control and SALS donors in the patient-derived motor neurons and postmortem spinal cord dataset sourced from Humphrey et al.^32^. Control data for correlations were generated by randomizing the order of the fold-change values for the spinal cord motor neuron test sets.

### Pharmacological testing

Wells were treated with DMSO, riluzole, edaravone, AMX0035 (sodium phenylbutyrate and taurursodiol), baricitinib (all from MedchemExpress) or memantine (from Tocris Bioscience) or left untreated at day 36 to a final concentration of 0–50 µM. A 50% change of the medium, containing DMSO or the study drugs at the specified concentration, was conducted every 3–4 days. Images were acquired and analyzed as described above, and the length of GFP-positive neurites was quantified using the average of four replicate wells per control or treatment group, with three images or fields of view per well. Data were expressed as days of rescue (treatment group LD_50_ − DMSO group LD_50_).

### Riluzole treatment transcriptional profiling

RNA was isolated at the LD_50_ for each donor, as mentioned above. RNA libraries were prepared using the Illumina Stranded mRNA Library Preparation Kit (Illumina) and sequenced on a NovaSeq 6000 sequencer (Illumina) to obtain approximately 20 million 150-bp paired-end reads per sample. RNAseq analysis was conducted as described above for the transcriptional profiling of patient-derived ALS motor neurons, with modified gene filtering (>2 counts per million in two samples) and design matrix (treatment only). Differentially expressed genes were identified between DMSO- and riluzole-treated patient-derived ALS motor neurons using adjusted *P* values of <0.05.

Correlation analysis was conducted on log_2_(fold-change) RNAseq data from differentially expressed genes identified in SALS donors (192 genes, false discovery rate < 0.5) and the riluzole treatment RNAseq dataset. Control data for correlations were generated by randomizing the order of the fold-change values for the riluzole treatment RNAseq test sets.

### Riluzole treatment calcium imaging

Motor neurons were cultured in 384-well plates and treated with DMSO or riluzole according to pharmacological testing. On day 42, the cultures were stained using the Rhod-3 calcium imaging kit (R10145, Thermo Fisher Scientific). Five wells, using two fields of view per well, were imaged, and calcium activity was captured at 10 Hz. All data underwent processing on the Python platform using Suite2p^78^ and analysis scripts as previously described^79^. In Suite2p (v0.14.2), regions of interest were selected using predefined settings and a classifier specifically designed for motor neurons. The changes in fluorescence intensity (Δ*F*/*F*) within each region of interest were calculated as the ratio of the fluorescence value to the baseline, which represented the median fluorescence value throughout the imaging session. Signals were filtered using a Savitzky–Golay filter before individual calcium events were detected as transients that were three times the s.d. of the baseline value. Calcium frequency and amplitude are presented as mean ± standard error. The data were analyzed using the Mann–Whitney *U* test.

### Clinically tested ALS drugs

A list of ALS clinical trials was generated using the search term ‘amyotrophic lateral sclerosis’ in the ClinicalTrials.gov database (www.clinicaltrials.gov/) and the World Health Organization’s International Clinical Trials Registry Platform (www.who.int/clinical-trials-registry-platform), or from previously published ALS clinical trials^6^. Interventional clinical trials of the type ‘drug’ were selected, excluding biological, procedural and device interventions. Further refinement was made to exclude non-disease-modifying interventions targeting nonmotor symptoms and diagnostic tracers. A total of 178 unique active ingredients were identified as being administered across the trials, including those given in drug combinations. Drugs that were not readily available or could not be tested in the iPS cell system—including nutritional interventions, proprietary compounds, complex herbal remedies, plasmids, antisense oligonucleotides and antibodies—were not assessed. The final list of tested drugs included 107 drugs (Supplementary Table 12).

Drug screening was conducted at a concentration of 2.5 μM, as described above, in duplicate wells from three 20× fields of view in two wells per donor culture. The length of GFP-positive neurites was quantified using the average of duplicate wells, with three images or fields of view per well. Drug screens (but not validation studies) were expressed relative to the average LD_50_ of all treatments screened per donor (based on the assumption that the average change will equal 0) to calculate the days of rescue and were discontinued 5 days after the LD_50_ of the DMSO controls to maximize throughput, limiting the maximum quantified rescue to 4–5 days.

### Statistical analysis

All quantification was conducted by blinded researchers using automated imaging and analysis pipelines (unless otherwise stated), with data represented as mean ± s.e.m. No statistical methods were used to predetermine sample sizes, but our sample sizes are similar to or larger than those reported in previous publications^15–18,23^. Normal data distribution and equal variance were formally tested (*n* > 3). Data collection and sample allocation were randomized. Data analysis was conducted using Prism 10 software (GraphPad) and the statistical tests described.

### Reporting summary

Further information on research design is available in the Nature Portfolio Reporting Summary linked to this article.

## Online content

Any methods, additional references, Nature Portfolio reporting summaries, source data, extended data, supplementary information, acknowledgements, peer review information; details of author contributions and competing interests; and statements of data and code availability are available at 10.1038/s41593-025-02118-7.

## Supplementary information

Supplementary Information Supplementary Note.

Reporting Summary

Supplementary Tables Supplementary Tables 1–14.

## Data Availability

Data supporting the findings of this study are available in the article and its supplementary information files. RNAseq data of riluzole-treated motor neurons were deposited into the Gene Expression Omnibus database (accession number GSE-TBA) and are available at the following URL: https://www.ncbi.nlm.nih.gov/geo/query/acc.cgi?acc=-TBA. Linked clinical, DNAseq and RNAseq data will be made available at the completion of the ID-MND initiative through the European Genome–phenome Archive (EGA) under controlled access and will be provided in accordance with the participants’ consent, institutional policies and relevant Australian laws. Postmortem RNAseq data are available at *Zenodo* (10.5281/zenodo.6385747)^80^.

## Code availability

All analyses were performed using freely available software as described in the Methods.
