# Supplementary material for: Large-scale drug screening in iPSC-derived motor neurons from sporadic ALS patients identifies a potential combinatorial therapy
Source: Nat Neurosci. 2025 Nov 24;29(1):40–52. doi: 10.1038/s41593-025-02118-7 (PMC12779551; doi:10.1038/s41593-025-02118-7)
Supplement: Supplementary file 1 — Supplementary Note. [file 41593_2025_2118_MOESM1_ESM.pdf]

nature neuroscience

Supplementary information

https://doi.org/10.1038/s41593-025-02118-7

# Large-scale drug screening in iPSC-derived motor neurons from sporadic ALS patients identifies a potential combinatorial therapy

In the format provided by the authors and unedited

Whole Genome Sequencing

Processing: FastQ-formatted sequencing data were assessed for quality using FastQC (v0.11.9). A total of N=109 MND and N=24 control met quality control cut-offs (N=3 excluded; 85, 185 and C113). Raw reads were aligned to human genome build 19 using the Burrows Wheeler Aligner (BWA-MEM v0.7.13-r1126) and sorted (sambamba, v0.7.1). Aligned reads were marked for duplicates using Picard tools and processed with GATK (v4.1.9.0) to recalibrate base quality scores. Variants for each sample were called with GATK and consolidated into a GVCF for joint-called SNPs and indels. The variants were outputted in VCF format and recalibrated using the GATK machine learning method with truth sensitivity filtering level = 99.0. The VCF files were converted into PLINK format and variants annotated with dbSNP reference IDs.

Sex, annotation and filtering: Sex checks were performed using a 0.15 reads ratio cut-off in Y vs. X chromosomes and the sex genotype-call confirmed to the recorded gender. The consolidated variant call file (VCF) was processed using ANNOVAR [1]. The table_annovar.pl function was used to integrate 17 databases (refGene, cytoBand, exac03, avsnp150, dbnsfp30a, clinvar_20220320, dbnsfp42c, intervar_20180118, gnomad211_genome, ALL.sites.2015_08, mcap13, regsnpintron, cadd13, fathmm, gwava, eigen). This enabled integration of gene and region-based annotation [2], pathogenic annotation for disease [3,4], population frequency annotation (1000g, ExAC, gnomAD) and functional prediction annotation (deleteriousness and conservation scoring (dbnsfp42c, mcap13, regsnpintron, cadd13, fathmm, gwava, eigen). Zoonomia bed files [5] were additionally matched to each genome position in the annotated VCFs for an additional conservation reference tool.

Pathogenic ALS mutations were identified and prioritized using a MAF frequency filter in reference populations of <9E-5 and a relevant gene list (described below). The MAF cut-off detects at least 90% of known ALS pathogenic SNVs curated in HGMD (n=318 ALS variants). To determine if the MAF cut-off frequency was consistent with disease in the population and genetic heterogeneity, the maximum credible allele frequency threshold was estimated [6]. Using the percentage of positive ALS cases with the *C9ORF72* HRE (7%) as most common genetic cause of ALS, a disease-specific MAF threshold of <1.8E-6 was identified. Comparison of the MAF filtered variant files identified little difference between the two thresholds, likely due to the size of the current reference population sample size, and the more conservative threshold was selected (<9E-5). To identify recessive ALS-causal variants a more relaxed MAF threshold of >0.01 was applied. The population maximum threshold was applied to ancestry specific reference populations in 1000g [7], ExAC [8] and gnomAD [9]. Rare variants were subsequently filtered with Func.refGene and “exonic” or “splicing” retained and “Synonymous” variants (ExonicFunc.refGene) or low-quality variant calls removed. The filtering steps were carried out for ALS genes known to cause disease (“Casual”, N=35). To ensure known ALS variants with a frequency above the MAF threshold were captured the VCF was also filtered for known ALS variants. Variants in genes annotated as causal for ALS were reported if they met the ACMG evidence criteria of “pathogenic” or “likely pathogenic” [10].

Gene list used to filter VCF

| | | | | |
|---|---|---|---|---|
| *ALS2* | *CHMP2B* | *HNRNPA1* | *SETX* | *TARDBP* |
| *ANG* | *DAO* | *KIF5A* | *SIGMAR1* | *TBK1* |
| *ANXA11* | *DCTN1* | *MATR3* | *SOD1* | *TIA1* |
| *ATXN2* | *ERBB4* | *NEFH* | *SPG11* | *TUBA4A* |
| *C9orf72* | *FIG4* | *NEK1* | *SQSTM1* | *UBQLN2* |
| *CCNF* | *FUS* | *OPTN* | *SS18L1* | *VAPB* |
| *CHCHD10* | *GLE1* | *PFN1* | *TAF15* | *VCP* |

iPSC Quality Control

SNP Array Analysis: Genotypes were generated using GenCall genotyper on the hg38 reference genome and converted to PLINK format using gcta. 759,993 genotypes were collected and SNPs converted to the positive strand using the GSAv2.0 strand designations (n=368,095) for comparison to ClinVar in downstream analyses. SNPs without mapping positions and multimapping SNP were removed (n=2,222). As CNV calling required independent SNP positions, duplicate variants were removed (n=2,990). Failed genotypes were filtered using the 'no call rate' method with a 0.15 threshold [11]. The average call rate was calculated and only autosomal variants were considered for SNP averages. 2,984 variants were removed resulting in 755,020 variants remaining.

Sample QC: Filtered SNPs were pruned by pairwise genetic correlation to create a set of independent variants for estimating relatedness. LD pruning was calculated using a window size of 1000 bp and 50 bp step with an r2 threshold of 0.5. Sample relatedness was assessed using the PIHAT estimator in PLINK. Pairs of samples with IBD scores suggestive of 1$^{st}$, 2$^{nd}$ and 3$^{rd}$ degree relatives were counted and the full pairwise IBD matrix was visualized using the ggplot2 package in R. Genders were confirmed using the '—checksex' flag in PLINK v1.9 [12] and gender and iPSC-blood pairing mismatches identified.

Karyotypic abnormalities were identified using virtual karyotyping with B Allele Frequency and Log R plots generated for all samples and ploidy, aberrant fraction and a confidence of karyotypic integrity quantified for all lines. SNPs were filtered by genotype missingness >0.05 and individual missingness >0.1. The post-QC genotyping rate was 99.27%. The inbreeding coefficient (F), genotype missingness and sample heterozygosity were used to estimate sample complexity (PLINK v1.9). The R package DNACopy was used to assess copy number changes [13,14] using the circular binary segmentation (CBS) algorithm to segment DNA copy number data and identify genomic regions with abnormal copy number [15,16]. Outliers defined as four standard deviations away from the next nearest point in a smoothing region were detected and smoothed. The samples were individually segmented into regions of estimated equal copy number using circular binary segmentation and a reference distribution estimated by permutation. Change-points due to local trends in the data were removed by taking all splits that were not at least three standard deviations apart. Confidence calls consisted of a positive or negative result in both the segment mean and the segment median. Regions smaller than

10,000 bp were removed and counts of gains and losses per probe estimated by the glFrequency function in the DNACopy package and ASCAT used to generate B-Allele frequency (BAF) plots per chromosome. Karyotypic abnormalities were identified by visual assessment of BAF plots and/or ploidy, aberrant cell fraction or karyotypic confidence calls deviating greater than 5% from expected. A single exception for a line with low karyotypic confidence was made based on low data quality. All karyotype aneuploidies (>10kb) were designated aberrant. In the subset of iPSCs displaying an abnormal karyotype, 10-20 monoclonal iPSC lines were generated per line and the clones subject to karyotyping. In 88% of donors, 3-5 karyotypically normal clones per line were identified and pooled to return the line to polyclonal status free of karyotypic abnormalities.

Quantification of Neurite Length

Image Analysis: Specific ImageJ [17] plugins were called in Knime as indicated below. Each image was pre-processed by calling imageJ's background subtraction method (rolling ball method, sliding slice size = 1 pixel) [18] to correct for uneven fluorescence illumination. Contrast-limited adaptive histogram equalization (CLAHE; blocksize = 19 pixels, histogram bins = 256, maximum slope = 8, version = fast) [19] was next applied to enhance local GFP contrast using imageJ's Enhance Local Contrast plugin. Each image then underwent two parallel paths of processing and segmentation to identify: i) neurites and ii) cell bodies. For neurite detection, ImageJ's Tubeness plugin (sigma = 2 pixels) [20]. Global thresholding with a manually set pixel intensity threshold value of 50 was applied, followed by connected component analysis and exclusion of objects smaller than 100 pixels. For cell body detection, a second CLAHE contrast adjustment was applied in Knime (contextual regions = 8, histogram bins = 256, maximum slope = 2.8), prior to local adaptive thresholding (method = Bernsen [21], span = 9 pixels, contrast threshold = 8700, neighbourhood type = spherical). Detected objects were cleaned via median filtering (span = 5), and objects with a radius of at least 11 pixels retained through a process of distance mapping, distance value thresholding (>= 5.5 pixels), Gaussian convolution (sigma = 3.5 by 3.5 pixels) and maxima detection – thus defining the centroids of cell bodies. To remove non-cellular background signals and approximate individual cells (cell bodies and neurites), Voronoi segmentation was performed on binarized neurite segments, using cell body centroids as seeds. To estimate individual cell body regions alone, Voronoi segmentation was applied to the median filtered objects, using cell body centroids as seeds, thus defining only cell body-like objects above the minimum radial threshold (11 pixels).

To quantify neurite lengths, per image, ImageJ's skeletonize plugin [22] was first applied to Voronoi segmented neurites. Image math was performed to subtract cell body regions from the skeletonized images, thus removing these objects (which include both live and dead cell bodies) from subsequent quantification of neurite length. Neurite length was calculated per object using ImageJ's Analyse Skeleton function.

<u>References</u>


1. Wang, K., Li, M. & Hakonarson, H. ANNOVAR: functional annotation of genetic variants from high-throughput sequencing data. *Nucleic acids research* **38,** e164; 10.1093/nar/gkq603 (2010).

2. Navarro Gonzalez, J. *et al.* The UCSC Genome Browser database: 2021 update. *Nucleic acids research* **49,** D1046-D1057; 10.1093/nar/gkaa1070 (2021).

3. Landrum, M. J. *et al.* ClinVar: improving access to variant interpretations and supporting evidence. *Nucleic acids research* **46,** D1062-D1067; 10.1093/nar/gkx1153 (2018).

4. Li, Q. & Wang, K. InterVar: Clinical Interpretation of Genetic Variants by the 2015 ACMG-AMP Guidelines. *American journal of human genetics* **100,** 267–280; 10.1016/j.ajhg.2017.01.004 (2017).

5. Zoonomia Consortium. A comparative genomics multitool for scientific discovery and conservation. *Nature* **587,** 240–245; 10.1038/s41586-020-2876-6 (2020).

6. Whiffin, N. *et al.* Using high-resolution variant frequencies to empower clinical genome interpretation. *Genetics in medicine : official journal of the American College of Medical Genetics* **19,** 1151–1158; 10.1038/gim.2017.26 (2017).

7. Auton, A. *et al.* A global reference for human genetic variation. *Nature* **526,** 68–74; 10.1038/nature15393 (2015).

8. Lek, M. *et al.* Analysis of protein-coding genetic variation in 60,706 humans. *Nature* **536,** 285–291; 10.1038/nature19057 (2016).

9. Wang, Q. *et al.* Landscape of multi-nucleotide variants in 125,748 human exomes and 15,708 genomes. *Nature communications* **11,** 2539; 10.1038/s41467-019-12438-5 (2020).

10. Richards, S. *et al.* Standards and guidelines for the interpretation of sequence variants: a joint consensus recommendation of the American College of Medical Genetics and Genomics and the Association for Molecular Pathology. *Genetics in medicine : official journal of the American College of Medical Genetics* **17,** 405–424; 10.1038/gim.2015.30 (2015).

11. Ritchie, M. E., Liu, R., Carvalho, B. S. & Irizarry, R. A. Comparing genotyping algorithms for Illumina's Infinium whole-genome SNP BeadChips. *BMC bioinformatics* **12,** 68; 10.1186/1471-2105-12-68 (2011).

12. Purcell, S. *et al.* PLINK: a tool set for whole-genome association and population-based linkage analyses. *American journal of human genetics* **81,** 559–575; 10.1086/519795 (2007).

13. Huber, W. *et al.* Orchestrating high-throughput genomic analysis with Bioconductor. *Nature methods* **12,** 115–121; 10.1038/nmeth.3252 (2015).

14. Seshan VE, O. A. DNAcopy: DNA copy number data analysis. R package. Available at https://www.bioconductor.org/packages/devel/bioc/vignettes/DNAcopy/inst/doc/DNAcopy.pdf (2016).

15. Olshen, A. B., Venkatraman, E. S., Lucito, R. & Wigler, M. Circular binary segmentation for the analysis of array-based DNA copy number data. *Biostatistics (Oxford, England)* **5,** 557–572; 10.1093/biostatistics/kxh008 (2004).

16. Venkatraman, E. S. & Olshen, A. B. A faster circular binary segmentation algorithm for the analysis of array CGH data. *Bioinformatics (Oxford, England)* **23,** 657–663; 10.1093/bioinformatics/btl646 (2007).

17. Schneider, C. A., Rasband, W. S. & Eliceiri, K. W. NIH Image to ImageJ: 25 years of image analysis. *Nature methods* **9,** 671–675; 10.1038/nmeth.2089 (2012).

18. Sternberg. Biomedical Image Processing. *Computer* **16,** 22–34; 10.1109/MC.1983.1654163 (1983).

19. Zuiderveld, K. VIII.5. - Contrast Limited Adaptive Histogram Equalization. In *Graphics Gems,* edited by P. S. Heckbert (Academic Press1994), pp. 474–485.

20. Sato, Y. *et al.* Three-dimensional multi-scale line filter for segmentation and visualization of curvilinear structures in medical images. *Medical image analysis* **2,** 143–168; 10.1016/s1361-8415(98)80009-1 (1998).

21. Bernsen J. Dynamic Thresholding of Gray Level Image. *Proceedings of International Conference on Pattern Recognition (ICPR '86),* 1251–1255 (1986).

22. Arganda-Carreras, I., Fernández-González, R., Muñoz-Barrutia, A. & Ortiz-De-Solorzano, C. 3D reconstruction of histological sections: Application to mammary gland tissue. *Microscopy research and technique* **73,** 1019–1029; 10.1002/jemt.20829 (2010).
