# Supplementary material for: Large-scale drug screening in iPSC-derived motor neurons from sporadic ALS patients identifies a potential combinatorial therapy
Source: Nat Neurosci. 2025 Nov 24;29(1):40–52. doi: 10.1038/s41593-025-02118-7 (PMC12779551; doi:10.1038/s41593-025-02118-7)
Supplement: Supplementary file 2 — Reporting Summary [file 41593_2025_2118_MOESM2_ESM.pdf]

Corresponding author(s): Bradley Turner

Last updated by author(s): May 21, 2025

Reporting Summary

Nature Portfolio wishes to improve the reproducibility of the work that we publish. This form provides structure for consistency and transparency in reporting. For further information on Nature Portfolio policies, see our Editorial Policies and the Editorial Policy Checklist.

Statistics

For all statistical analyses, confirm that the following items are present in the figure legend, table legend, main text, or Methods section.

n/a	Confirmed	
☐	☒	The exact sample size (*n*) for each experimental group/condition, given as a discrete number and unit of measurement
☐	☒	A statement on whether measurements were taken from distinct samples or whether the same sample was measured repeatedly
☐	☒	The statistical test(s) used AND whether they are one- or two-sided<br>*Only common tests should be described solely by name; describe more complex techniques in the Methods section.*
☐	☒	A description of all covariates tested
☐	☒	A description of any assumptions or corrections, such as tests of normality and adjustment for multiple comparisons
☐	☒	A full description of the statistical parameters including central tendency (e.g. means) or other basic estimates (e.g. regression coefficient) AND variation (e.g. standard deviation) or associated estimates of uncertainty (e.g. confidence intervals)
☐	☒	For null hypothesis testing, the test statistic (e.g. *F*, *t*, *r*) with confidence intervals, effect sizes, degrees of freedom and *P* value noted<br>*Give P values as exact values whenever suitable.*
☒	☐	For Bayesian analysis, information on the choice of priors and Markov chain Monte Carlo settings
☒	☐	For hierarchical and complex designs, identification of the appropriate level for tests and full reporting of outcomes
☐	☒	Estimates of effect sizes (e.g. Cohen's *d*, Pearson's *r*), indicating how they were calculated

Our web collection on statistics for biologists contains articles on many of the points above.

Software and code

Policy information about availability of computer code

Data collection	ZEISS Celldiscoverer 7 Microscope, ZEN 2.6 (Blue edition) Carl Zeiss Microscopy GmbH,2018<br>ViiA™ 7 Real-Time PCR System, QuantStudio TM Real-Time PCR System 1.3<br>Opera Phenix Plus High-Content Screening System, Harmony 5.1.2167.302<br>Illumina iScan SNP Genotyping, GenomeStudio 2.0.3 with Genotyping module<br>NovaSeq 6000 System, NovaSeq Control Software 1.7.5, Real Time Analysis 3.4.4, DRAGEN<br>BCL Convert 07.021.624.3.10.8
Data analysis	SNP Genotyping Analysis: PLINK (1.9), R (3.6), Bioconductor (3.10)<br>Whole genome sequencing: FastQC (0.11.9), Burrows Wheeler Aligner (BWA-MEM 0.7.13-r1126), sambamba (0.7.1), GATK (4.1.9.0), Expansion hunter (4)<br>Image Processing and Neurite Quantification: Knime (4.6.3), ImageJ (1.53), GraphPad Prism (9.1), Suite2p (0.14.2).<br>Whole transcriptome sequencing: R (4.2.2), Rsubread (2.12.2) , biomaRt (2.54.0), edgeR (3.40.1) , variancePartition (1.34.0), limma (3.54.0) , Gplots (3.1.3), clusterProfiler (v4.8.3) , RegTools (v1.0.0), leafercutter (v0.2.9).

For manuscripts utilizing custom algorithms or software that are central to the research but not yet described in published literature, software must be made available to editors and reviewers. We strongly encourage code deposition in a community repository (e.g. GitHub). See the Nature Portfolio guidelines for submitting code & software for further information.

Data

Policy information about availability of data

All manuscripts must include a data availability statement. This statement should provide the following information, where applicable:

- Accession codes, unique identifiers, or web links for publicly available datasets
- A description of any restrictions on data availability
- For clinical datasets or third party data, please ensure that the statement adheres to our policy

Data supporting the findings of this study are available within the paper and its Supplementary Information. Sequencing data are available through the European Genome-phenome Archive (EGA) under controlled access and will be provided in accordance with the participant's consent, institution policy and relevant Australian laws. Post-mortem RNAseq data is available at Zendo 10.5281/zenodo.6385747

Research involving human participants, their data, or biological material

Policy information about studies with human participants or human data. See also policy information about sex, gender (identity/presentation), and sexual orientation and race, ethnicity and racism.

Reporting on sex and gender	The sex of participants was assigned using SNP analysis (male n=93, female n=43) and pooled in all analysis.
Reporting on race, ethnicity, or other socially relevant groupings	No race, ethnicity groupings of participants.
Population characteristics	Age, ALS disease status, sex, and ALS genetic variants reported for all participants
Recruitment	Participants were recruited though advertisements in ALS community support groups newsletters and in specialist ALS medical clinics. Participants did not receive compensation for their time.
Ethics oversight	The collection of human tissue, clinical and demographic information was approved by the University of Melbourne Human Research Ethics Committee (ID 1749960).

Note that full information on the approval of the study protocol must also be provided in the manuscript.

Field-specific reporting

Please select the one below that is the best fit for your research. If you are not sure, read the appropriate sections before making your selection.

☒ Life sciences ☐ Behavioural & social sciences ☐ Ecological, evolutionary & environmental sciences

For a reference copy of the document with all sections, see nature.com/documents/nr-reporting-summary-flat.pdf

Life sciences study design

All studies must disclose on these points even when the disclosure is negative.

Sample size	The iPSC library sample size was selected to match the size of a well-powered mid-phase clinical trial (n=136), providing broad patient coverage to encompass clinical and molecular heterogeneity in the patient population. Phenotypic screening and transcriptional profiling of the library included all donors, exceeding the standard in the field.<br>Analysis of monogenic donors was limited by the availability of lines, and phenotypic assessment conducted using 3 biological replicates of individual donors and/or coverage across multiple impacted genes/individuals as appropriate.<br><br>Drug screening/testing was conducted using 7-16 sporadic ALS donors to capture potential heterogeneity in drug responses, exceeding the current standard in the field.
Data exclusions	Neurite quantification: Donors/wells with insufficient motor neurons for accurate quantification were excluded from the analysis. Criteria for exclusion were developed during the protocol optimization and implemented in subsequent assays as described in the methods.<br><br>Transcriptomic Analysis: Donors with insufficient RNA quality/quantity for accurate profiling or identified as failed differentiations were excluded from transcriptomic analysis. Criteria for exclusion were developed during the project and implemented in subsequent profiling as described in the methods.<br><br>Drug Testing/Screening: Donors with LD50 >46 were deemed not to provide a sufficient phenotype for accurate drug testing and were excluded from the analysis. Criteria for exclusion were developed during the project and implemented in subsequent assays as described in the methods.
Replication	All experiments were conducted with multiple biological replicates, or across multiple donors/individuals to capture heterogeneity in the patient population. Significant differences in motor neuron health were successfully validated in independent experiments and by cross-validation with direct quantification of motor neuron survival.

Randomization	Participants were randomly allocated into batches containing proportional representation of healthy control and disease cases for screening.
Blinding	All samples were anonymised and data acquisition and data analysis automated or conducted by blinded investigators.

Reporting for specific materials, systems and methods

We require information from authors about some types of materials, experimental systems and methods used in many studies. Here, indicate whether each material, system or method listed is relevant to your study. If you are not sure if a list item applies to your research, read the appropriate section before selecting a response.

Materials & experimental systems

n/a	Involved in the study
☐	☒ Antibodies
☐	☒ Eukaryotic cell lines
☒	☐ Palaeontology and archaeology
☒	☐ Animals and other organisms
☐	☒ Clinical data
☒	☐ Dual use research of concern
☒	☐ Plants

Methods

n/a	Involved in the study
☒	☐ ChIP-seq
☒	☐ Flow cytometry
☒	☐ MRI-based neuroimaging

Antibodies

Antibodies used

ChAT (Anti-Choline Acetyltransferase Antibody, Merck, AB144P, polyclonal, 3520940)
GFAP (GFAP Monoclonal Antibody, Thermo Fisher, 13-0300, 2.2B10, WL34181)
GFP (GFP Antibody, R&D, mab42401, 454505, CHYN0119061)
HB9/MNX1 (MNR2/HB9/MNX1 MNR2 Antibody, DEVSTU, 81.5C10-S, 81.5C10, 2/14/19)
IBA1 (Anti IBA1 Polyclonal Antibody, Fujiflim WakoPure Chemical Corporation, 019-19741, polyclonal, LEE6003)
βIII Tubulin (Anti-β Tubulin Antibody (F-1), PROMEGA, G7121, 5G8, 0000530908)
TDP43 (TDP-43 Polyclonal antibody, Proteintech, 10782-2-AP, polyclonal, 91859)

Validation

ChAT (validated by supplier (AB144P), the Antibody registry (RRID: AB_2079751), and in literature (429 citations in SciCrunch).
GFAP (validated by supplier (13-0300), the Antibody registry (RRID: AB_2532994), and in literature (44 citations in SciCrunch).
GFP (validated by supplier (mab42401), and in literature (1 citations in Google Scholar).
HB9/MNX1 (validated by supplier (81.5C10-S), the Antibody registry (RRID: AB_2145209), and in literature (64 citations in SciCrunch).
IBA1 (validated by supplier (019-19741), the Antibody registry (RRID: AB_839504), and in literature (705 citations in SciCrunch).
βIII Tubulin (validated by supplier (G7121), the Antibody registry (RRID: AB_430874), and in literature (49 citations in SciCrunch).
TDP43 (validated by supplier (10782-2-AP), the Antibody registry (RRID: AB_430874), and in literature (89 citations in SciCrunch)

Eukaryotic cell lines

Policy information about cell lines and Sex and Gender in Research

Cell line source(s)	Primary fibroblasts from human donors were reprogrammed using non-integrating vectors to generate iPSC lines. The sex of all donors was determined by genetic analysis and is reported in the supplementary data.
Authentication	Cell line authentication was conducted by pairwise comparisons to donor blood. Genetic profiles were established for all cell lines to allow future authentication.
Mycoplasma contamination	All iPSC lines were tested and confirmed mycoplasma negative.
Commonly misidentified lines (See ICLAC register)	*Name any commonly misidentified cell lines used in the study and provide a rationale for their use.*

Clinical data

Policy information about clinical studies

All manuscripts should comply with the ICMJE guidelines for publication of clinical research and a completed CONSORT checklist must be included with all submissions.

Clinical trial registration	Not applicable
Study protocol	Not applicable
Data collection	Patient skin biopsies and blood were collected in a clinical setting between December 2017 and August 2018 for iPSC generation and pairwise iPSC quality control/genetic analysis respectively.

Outcomes	Not applicable
